# Mechanistic Insight into the Precursor Chemistry of ZrO_2_ and HfO_2_ Nanocrystals; towards Size-Tunable Syntheses

**DOI:** 10.1021/jacsau.1c00568

**Published:** 2022-03-09

**Authors:** Rohan Pokratath, Dietger Van den Eynden, Susan Rudd Cooper, Jette Katja Mathiesen, Valérie Waser, Mike Devereux, Simon J. L. Billinge, Markus Meuwly, Kirsten M. Ø. Jensen, Jonathan De Roo

**Affiliations:** † Department of Chemistry, University of Basel, Mattenstrasse 24, BPR 1096, Basel 4058, Switzerland; ‡ Department of Chemistry, University of Copenhagen, Universitetsparken 5, Copenhagen 2100, Denmark; § Department of Chemistry, University of Basel, Klingelbergstrasse 80, Basel 4056, Switzerland; ∥ Applied Physics and Applied Mathematics Department, Columbia University, New York, New York 10027, United States; ⊥ Condensed Matter Physics and Material Science Department, Brookhaven National Laboratory, Upton, New York 11973, United States

**Keywords:** metal oxide, nanoparticle, non-aqueous, PDF, DFT, NMR, surfactant

## Abstract

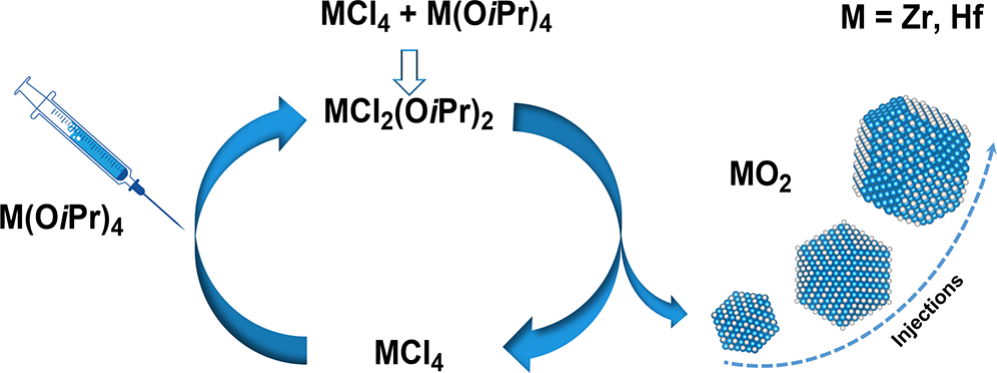

One can nowadays readily generate monodisperse colloidal nanocrystals, but a retrosynthetic analysis is still not possible since the underlying chemistry is often poorly understood. Here, we provide insight into the reaction mechanism of colloidal zirconia and hafnia nanocrystals synthesized from metal chloride and metal isopropoxide. We identify the active precursor species in the reaction mixture through a combination of nuclear magnetic resonance spectroscopy (NMR), density functional theory (DFT) calculations, and pair distribution function (PDF) analysis. We gain insight into the interaction of the surfactant, tri-*n*-octylphosphine oxide (TOPO), and the different precursors. Interestingly, we identify a peculiar X-type ligand redistribution mechanism that can be steered by the relative amount of Lewis base (L-type). We further monitor how the reaction mixture decomposes using solution NMR and gas chromatography, and we find that ZrCl_4_ is formed as a by-product of the reaction, limiting the reaction yield. The reaction proceeds via two competing mechanisms: E1 elimination (dominating) and S_N_1 substitution (minor). Using this new mechanistic insight, we adapted the synthesis to optimize the yield and gain control over nanocrystal size. These insights will allow the rational design and synthesis of complex oxide nanocrystals.

## Introduction

Group 4 metal oxides (titania, zirconia, and hafnia) exhibit interesting material properties such as high dielectric constant, wide bandgap, chemical and thermal resistance, fracture toughness, and high refractive index. Because of their high dielectric constant, ZrO_2_ and HfO_2_ are considered as a potential replacement for SiO_2_ (gate insulator) in field-effect transistors.^1,2^ Furthermore, the mixed metal oxide Hf_1–*x*_Zr*_x_*O_2_ is a ferroelectric material of interest.^3,4^ Nanocrystals of the group 4 oxides have many applications such as superconducting nanocomposites,^5,6^ optical nanocomposites,^7−9^ dentistry,^10^ (photo)catalysis,^11−13^ coatings,^14−16^ and X-ray computed tomography contrast agents.^17^ Colloidal stability and control over nanocrystal size is a prerequisite for many of these technologies. Consequently, synthetic procedures to produce colloidally stable group 4 oxide nanocrystals are actively researched.^18^

Non-aqueous, bottom-up syntheses have been very successful at producing crystalline metal oxide nanoparticles. On the one hand, there are surfactant-free syntheses where benzyl alcohol (and benzylamine) have yielded nanocrystals that could often be dispersed in an organic solvent by the post-synthetic addition of ligands.^19−25^ On the other hand, surfactant-assisted syntheses generally provide more control over the final crystal size,^26,27^ phase,^28,29^ composition,^30,31^ and shape.^32−36^ Dopants are also regularly incorporated.^37,38^ A particularly general approach was inspired by the non-aqueous gel formation of titania, as reported by Vioux et al. Titanium chloride reacts with titanium isopropoxide to titania gels at 100 °C.^39^ When tri-*n*-octylphosphineoxide (TOPO) is added as ligand/solvent (bp = 408 °C), titania nanocrystals are obtained at 300 °C.^27^ The reaction is believed to produce isopropyl chloride as a co-product (no data provided) and the proposed mechanism is an S_N_1 nucleophilic substitution (based on the increased reactivity in the series; titanium methoxide, ethoxide, *iso*-propoxide, and *tert*-butoxide). The same method could be generalized to zirconia, hafnia, and solid solutions of zirconia and hafnia.^26,29,30,36,40^ Since TOPO is the only surfactant present, it was generally assumed that TOPO is the ligand bound to the nanocrystal surface. However, we recently reported that during the nanocrystal synthesis, TOPO undergoes thermal decomposition into di-*n*-octylphosphinic acid and pyrophosphonate. Given that these decomposition products have a higher affinity for the nanocrystal surface than TOPO itself, the final surface chemistry is a complex mixture of the three species.^41^ Octene is another by-product of TOPO’s decomposition, and is typically observed refluxing at the reaction temperature.^42^

**Scheme 1:**
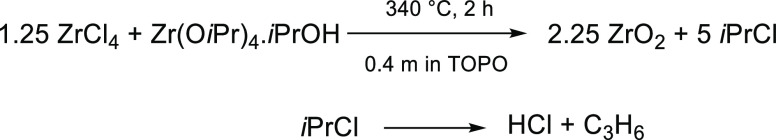
Proposed Reaction Scheme of Zirconium Chloride and Zirconium Isopropoxide Isopropanol Complex, Reacting towards ZrO_2_ Nanocrystals, According to Joo et al.^26^

Compared to titania and hafnia, zirconia nanocrystals produced in TOPO are the most monodisperse, and feature the best colloidal stability. It is worth examining the proposed reaction scheme more closely (Scheme 1).^26^ Slightly more zirconium chloride (1.25 equiv) was added to compensate for the isopropanol molecule coordinated to the zirconium isopropoxide precursor. Isopropyl chloride and propene were determined to be by-products via gas chromatography. Presumably, isopropyl chloride is formed in the same way as for titania (S_N_1 nucleophilic substitution). The isopropyl chloride is believed to undergo dehydrohalogenation to propene. Note that it is also possible to replace ZrCl_4_ with its tetrahydrofuran complex (ZrCl_4_·2THF) to improve the solubility of the chloride.^41^ Using zirconium chloride, the synthesis produces 4 nm nanocrystals while 3 nm nanocrystals are obtained with zirconium bromide. However, no further size tuning has been reported. This is a clear limitation of the current state-of-the-art, especially when compared to the exquisite size control exerted in the field of semiconductor quantum dots (e.g., PbS).^43^ Finally, the yield of this reaction seems limited to about 50%.^15,41^

In this report, we aim at obtaining deeper insight into the precursor chemistry of these nanocrystal reactions, and we aim at introducing size tunability. We first analyze the speciation of the precursors using solution ^1^H and ^31^P NMR spectroscopy, supported by density functional theory (DFT) calculations to better understand their interaction with the coordinating solvent, TOPO. Second, by using control experiments and independent synthesis of the proposed species, we identify the actual reaction precursors; mixed chloroalkoxides. Furthermore, we reveal an alternative mechanism based on E1 elimination, which is happening in parallel to the earlier proposed S_N_1 mechanism and we thus come to an overall, fully balanced reaction equation. We confirm the formation of ZrCl_4_ as a by-product by NMR and X-ray total scattering studies. Finally, using our insight into the reaction mechanism, we then increase the yield of the reaction, while also providing a way to tune the final nanocrystal size.

## Results and Discussion

### Interaction of ZrCl_4_ with TOPO

**Figure 1:**
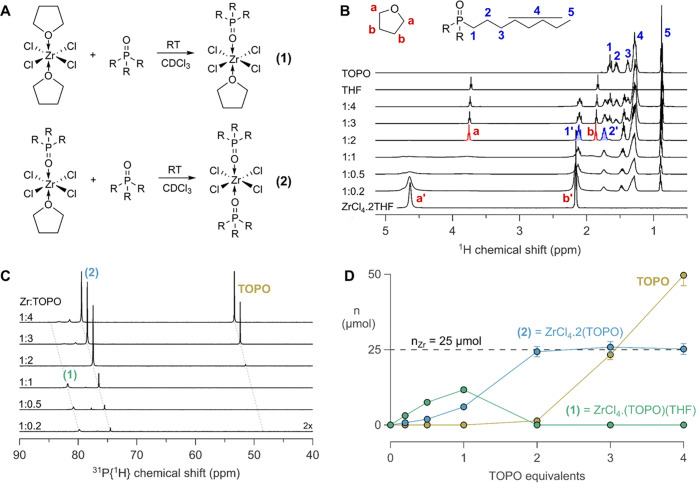
(A) Scheme for the reaction of ZrCl_4_·2THF with TOPO. (B) ^1^H NMR of the titration of a solution of 0.05 M ZrCl_4_·2THF in CDCl_3_ with increasing equivalents of TOPO. The latter is added as a 0.5 M solution, gradually diluting the zirconium complex. (C) ^31^P NMR of the same titration. The spectrum for 0.2 equiv was amplified twofold to observe the resonances more clearly. The spectra have a relative x-offset of 1 ppm with respect to each other. (D) The different TOPO species over the course of the titration. The total amount of Zr in the sample was 25 μmol.

We first investigate the speciation of the precursors and their interaction with the coordinating solvent; tri-*n*-octylphosphine oxide (TOPO). Starting from the soluble THF complex of zirconium chloride in CDCl_3_, we gradually add TOPO (as a 0.5 M solution in CDCl_3_) and monitor the reaction by ^1^H and ^31^P NMR (Figure 1). The resonances of THF bound to ZrCl_4_ (**a′** and **b′**) are shifted to higher ppm values compared to free THF (**a** and **b**), see Figure 1B. Upon addition of TOPO, we observe a decrease in the **a′** and **b′** resonances, and resonance **a** shows up initially as a very broad resonance around 4 ppm and then sharpens and shifts to 3.74 ppm. We also clearly observe the growth of resonance **1′** (2.11 ppm, TOPO bound to ZrCl_4_) up to 2 equiv of TOPO. It is only by the third TOPO equivalent, that resonance **1** of free TOPO is observed. These observations indicate that TOPO irreversibly displaces THF from the zirconium chloride complex, in a Lewis base exchange reaction. The stoichiometry is corroborated by the relative integrals of free THF and bound TOPO (Figure S1). The ^31^P NMR spectrum yields even more insight into the speciation (Figure 1C). The resonance at 74.8 ppm is the main product after the addition of 2 equiv TOPO and is assigned to the double TOPO adduct of zirconium chloride **(2)**, see Figure 1. This is verified by the direct synthesis of **(2)** from ZrCl_4_ and 2 equiv of TOPO (see Figure S2). In C_6_D_6_, **(2)** appears at 73 ppm (Figure S3). A Job plot of **(2)** also confirms the stoichiometry of two TOPO molecules per zirconium center (see Figure S4).^44^

The resonance at 79.8 ppm is assigned to complex **(1)** based on its higher chemical shift (indicating a higher Lewis acidity of the metal center).^45−47^ After adding the third TOPO equivalent, free TOPO is observed at 48 ppm in the ^31^P NMR spectrum, consistent with our analysis of the ^1^H NMR spectrum (Figure 1B). Based on the integrals of the ^31^P NMR spectrum, we calculate the amount of each species present during the titration (Figure 1D). It is clear that **(1)** is a transient species *en route* to **(2)**. Note that **(2)** is formed together with **(1)**, even at low equivalents, indicating that the second exchange is competitive with the first exchange. After 2 equiv of TOPO added, all ZrCl_4_ is coordinated with two TOPO ligands. Indeed, TOPO is a much stronger Lewis base than THF according to the SbCl_5_ affinity scale (592 vs 368.2 kJ/mol).^48^ This further underscores our assignment of **(1)** since the weakly basic THF leaves the zirconium center in **(1)** more Lewis acidic, causing a higher ^31^P NMR chemical shift.

**Figure 2:**
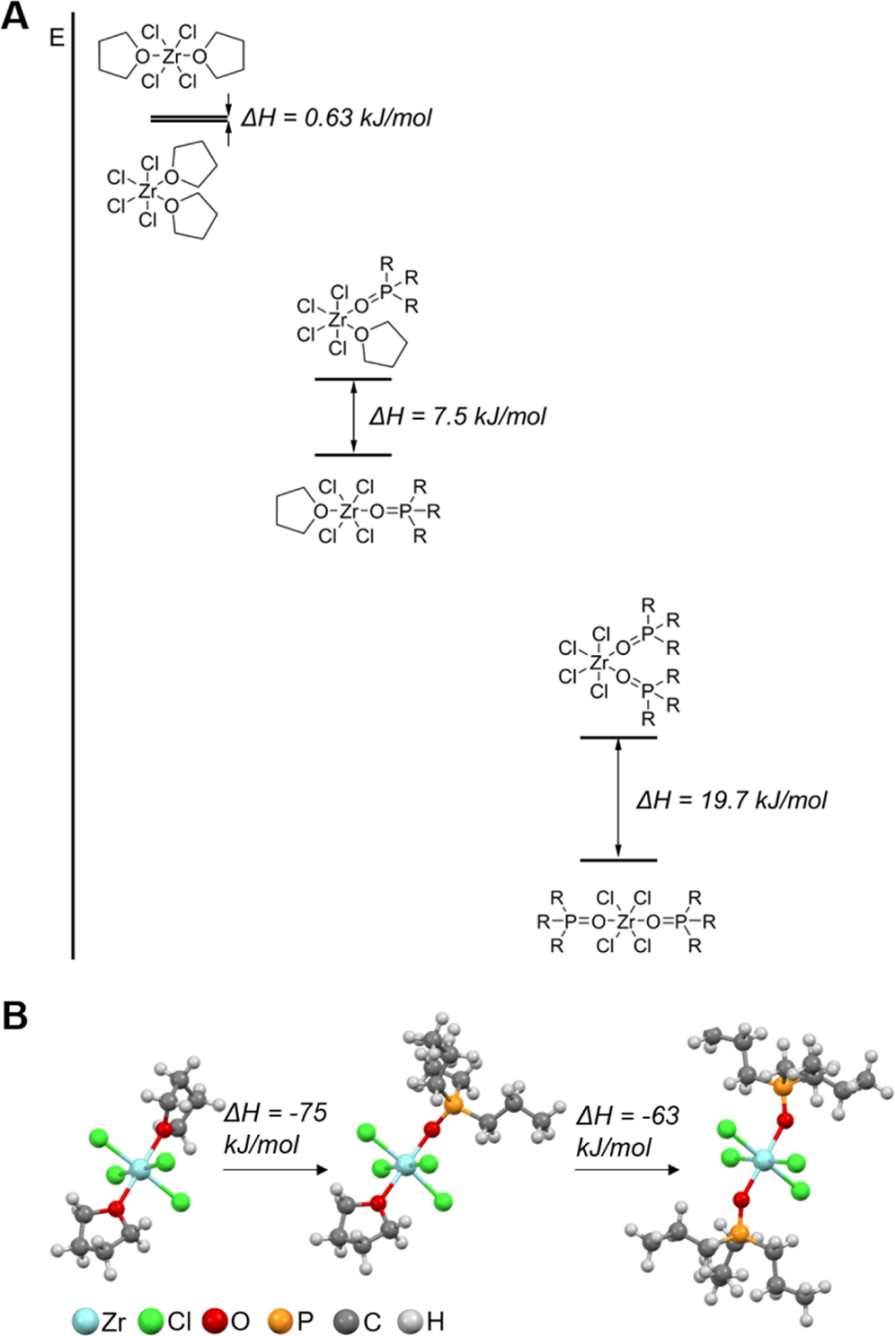
(A) Scheme showing the exchange reaction between the ZrCl_4_·2THF and tri-*n*-propylphosphine oxide ligands, comparing both cis and trans structures (R = propyl chain). (B) Δ*H* of the exchange reactions for the trans complexes and their corresponding optimized structures at the B3LYP/ aug-cc-pVDZ level of theory.

Having established the stoichiometry of the final complex **(2)**, we turn to its geometry. Single crystal data shows that ZrCl_4_·2THF has a cis geometry in the solid-state,^49^ however, TOPO is more sterically demanding. Unfortunately, we were unsuccessful in crystallizing **(2)** or any of its shorter chain derivatives with, e.g., triethylphosphine oxide. We also deemed triphenylphosphine oxide not a representative substitute, given its larger Tolmann cone angle and lower basicity, compared to trialkylphosphines.^50^ Therefore, using calculations at the density functional theory (B3LYP) level of theory, the energy minimized structure for the ZrCl_4_·2THF, ZrCl_4_·(THF)(TPPO), and ZrCl_4_·2TPPO complexes were determined with tri-*n*-propylphosphine oxide (TPPO) instead of TOPO as the ligand. Comparing the optimized structures of *cis* and *trans* ZrCl_4_·2THF, we find only a negligible energy difference (0.6 kJ/mol), see Figure 2. Since the cis isomer has a dipole moment, dipole-dipole interactions might explain the observation of the cis isomer in the solid state crystal structure.^49^ Comparing the bond lengths in the latter (Zr-Cl: 2.39 and 2.42 Å; Zr-O: 2.23 and 2.24 Å) with the DFT optimized structure (Zr-Cl: 2.39 and 2.45 Å; Zr-O: 2.34 Å), we find reasonable agreement, thus providing confidence in the quantum chemical calculations. Upon substitution of THF for TPPO, we find that the energy difference between the isomers progressively increases (Figure 2). The trans conformer is more stable by 7.5 and 20 kJ/mol for the single and double exchanged structures, respectively. Taking the trans complexes for each stoichiometry as the reference, we calculate the change in enthalpy (Δ*H*) for the exchange reactions. The first exchange accounts for −75 kJ/mol and the second is only slightly less exothermic (−63 kJ/mol) and thus competitive with the first exchange, which is consistent with our NMR experiments.

### Interaction of Zr(O*i*Pr)_4_·*i*PrOH with TOPO

**Figure 3:**
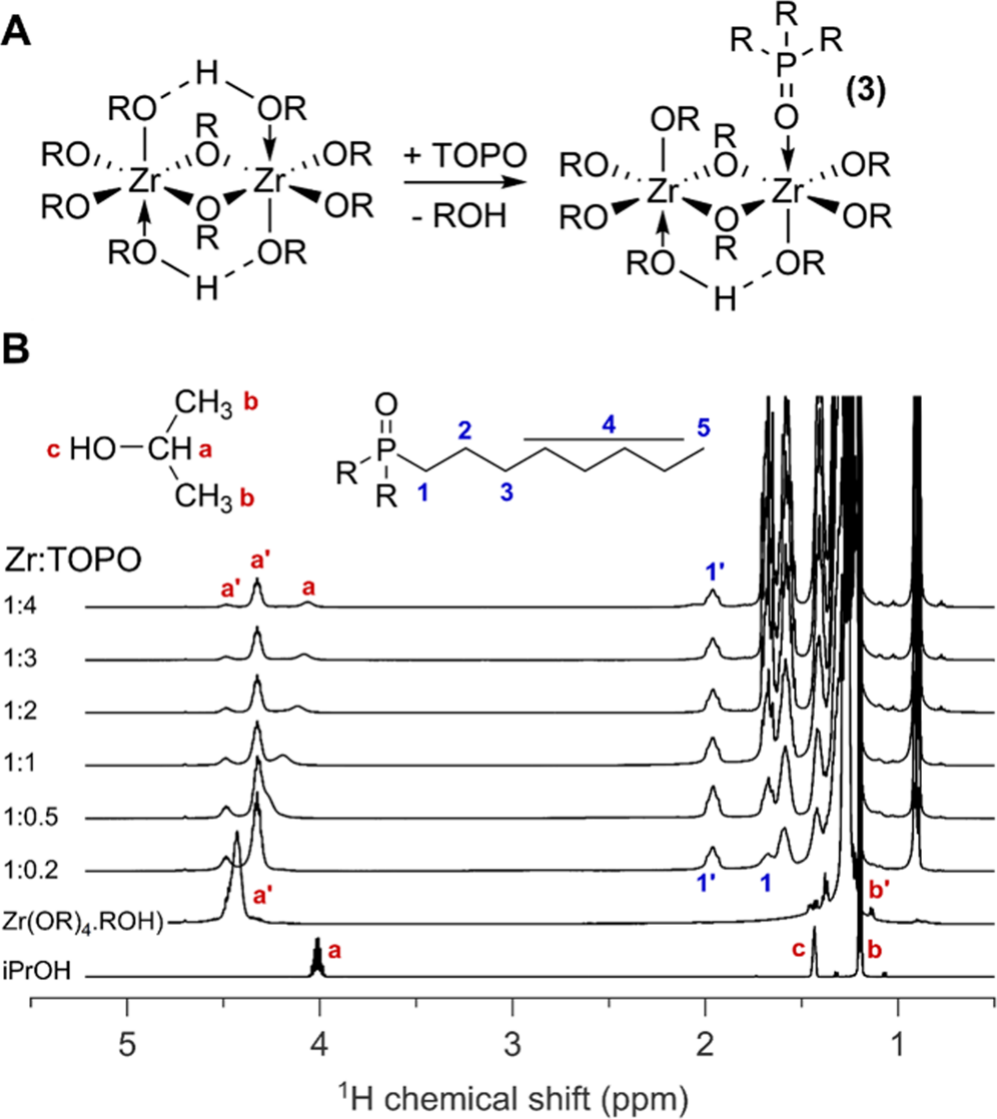
(A) Scheme for the reaction of Zr(O*i*Pr)_4_·*i*PrOH with TOPO. (B) ^1^H NMR of the titration of a solution of 0.05 M Zr(O*i*Pr)_4_·*i*PrOH in CDCl_3_ with increasing equivalents of TOPO relative to Zr. TOPO is added as a 0.5 M solution, gradually diluting the zirconium complex. The total amount of Zr in the sample was 25 μmol.

We find an interesting contrast by performing the same experiment with the zirconium isopropoxide isopropanol complex (Figure 3). It was previously shown that this complex is a dimer, in the solid-state and in solution.^51^ In the ^1^H NMR spectrum of Zr(O*i*Pr)_4_·*i*PrOH, we find resonance **a′** of isopropanol (CH) as a single broad peak at 4.4 ppm, while it should appear at 4.1 ppm for pure isopropanol (Figure 3). This is consistent with the coordination of isopropoxide/isopropanol to Zr and indicates that all CH protons have a roughly similar chemical environment. We also observe a very broad resonance at 5.2 ppm which integrates to one, when the CH integral integrates to five (Figure S5). We assign this resonance to the alcoholic proton (OH) of the coordinated isopropanol. The resonance is shifted considerably compared to free isopropanol in CDCl_3_ (1.43 ppm). Indeed, coordination of isopropanol with zirconium renders the alcohol proton more acidic.

Upon addition of 0.2 equiv of TOPO relative to Zr, resonance **a′** splits in two (1:4 ratio). We assign the least intense resonance at 4.5 ppm to the bridging isopropoxide in the dimer. Upon the addition of more TOPO, the more intense resonance splits again in two. Resonance **a** at 4.1 ppm accounts for 20% of the total CH integral and is assigned to isopropanol. Isopropanol is in a fast exchange between a free and a coordinated state since resonance **a** sharpens up and shifts upon gradual TOPO addition. The resonance **a′** at 4.3 ppm is assigned to non-bridging isopropoxide. We observe the bound TOPO resonance **1′** at 1.96 ppm. This value is slightly lower than the 2.11 ppm found for TOPO bound to ZrCl_4_ and is consistent with the lower expected Lewis acidity of Zr(O*i*Pr)_4_. In the ^31^P NMR spectrum, we find a single species **(3)** at 63 ppm, apart from the resonance of free TOPO (Figure S6A). We hypothesized three possible structures for **(3)**, which differ in their TOPO-to-zirconium stoichiometry (Figure S7). A job plot of **(3)** is most consistent with a Zr:TOPO stoichiometry of 1:0.5 (Figure S8), and we thus propose a dimer with a single TOPO ligand, see Figure 3A. This species appears not to be thermodynamically favored and is in equilibrium with free TOPO. Even at only 0.2 TOPO equivalents, we observe free TOPO in both the ^1^H and ^31^P NMR spectrum. Integration of the ^31^P NMR shows that only a small amount of **(3)** is formed (Figure S6B). Even when TOPO is added in excess (4 equiv), only 9 μmol of **(3)** is formed, while the total amount of Zr dimer is 12.5 μmol. This is a surprising result since isopropanol is even slightly less Lewis basic than THF (Figure S9) and thus much weaker than TOPO. However, DFT calculations show that the exchange of isopropanol for TPPO is endothermic with the least positive Δ*H* for our hypothesized structure **(3)**, see Figure S10. It is thus clear that intramolecular hydrogen bonding stabilizes the Zr(O*i*Pr)_4_·*i*PrOH dimer.

### Interaction of Precursor Mixtures with TOPO

In the literature, a 1.25:1 mixture of ZrCl_4_ and Zr(O*i*Pr)_4_·*i*PrOH is typically used to synthesize zirconia nanocrystals.^15,26,41^ This approach assumes that the extra 0.25 equiv ZrCl_4_ react with the coordinated isopropanol of Zr(O*i*Pr)_4_·*i*PrOH. Here we test the validity of that hypothesis. Mixing 1.25 equiv of ZrCl_4_·2THF with 1 equiv of Zr(O*i*Pr)_4_·*i*PrOH and titrating it with TOPO (Figure S11), we observe unambiguously the resonance of free isopropanol at 4.1 ppm, indicating that the coordinated isopropanol does not react with the excess ZrCl_4_. We rationalize this as follows. It is known that metathesis occurs by mixing metal complexes, randomly distributing the ligands over the available metal centers.^53,54^ Therefore, we can write
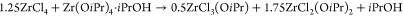
1We also know that ZrCl_4_ only reacts with a single equivalent of alcohol, even when the alcohol is in excess.^55^
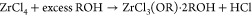
2This means that ZrCl_3_(OR) is unreactive to alcohols. Therefore, the above ZrCl_3_(O*i*Pr) and ZrCl_2_(O*i*Pr)_2_ (produced from mixing the reagents) do not react further with isopropanol.

**Figure 4:**
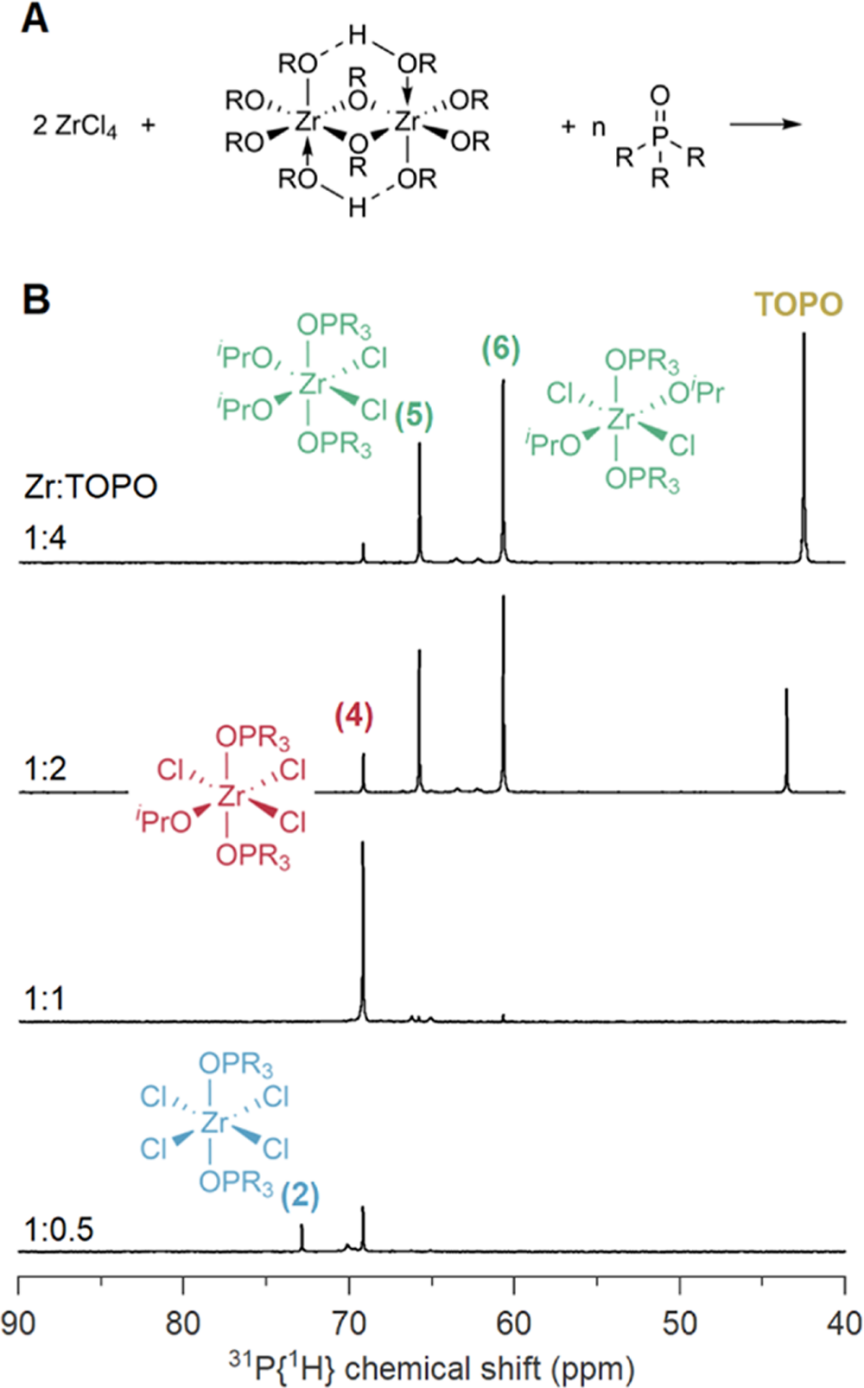
(A) Reaction scheme for the titration of a 1:1 mixture of ZrCl_4_:Zr(O*i*Pr)_4_·*i*PrOH with TOPO in C_6_D_6_ at room temperature. (B) The ^31^P NMR spectra of the titration. The ratio of Zr to TOPO is indicated in the figure.

To gain further insight into the speciation, we turned to ^31^P NMR. Unfortunately, the ^31^P NMR spectrum of the precursor mixture in CDCl_3_ is highly complex (Figure S11), most likely due to the hydrogen bonding capabilities of deuteroform. The spectrum in deuterated benzene is more convenient to analyze. Again, we observe the free isopropanol in the proton NMR spectrum, integrating for one while the isopropoxide resonance integrates for four (Figure S12). Focusing below only on equimolar mixtures, we titrated the precursor mixture with TOPO. In the ^31^P NMR spectrum (Figure 4), we observe the previously identified **(2)** at 0.5 TOPO equivalents. At 1 equiv TOPO, a single species **(4)** is observed at 69 ppm, which we identified as ZrCl_3_(O*i*Pr)·2TOPO. This is corroborated by the direct synthesis of **(4)**, either by reacting ZrCl_4_ with dry isopropanol (eq 2) or by reacting Zr(O*i*Pr)_4_·*i*PrOH with acetyl chloride (eq 3),^56^ see Figure S13A. The Job plot indicates again 2 TOPO ligands per zirconium center (Figure S13B,C).
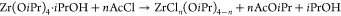
3

Upon addition of more TOPO, the resonance of **(4)** decreases again in intensity and new species **(5)** and **(6)** appear (at 65.7 and 60.7 ppm), which we assign as the two isomers of ZrCl_2_(O*i*Pr)_2_·2TOPO and which are the expected products of an equimolar mixture. The chemical identity of ZrCl_2_(O*i*Pr)_2_·2TOPO is again corroborated by the direct synthesis of ZrCl_2_(O*i*Pr)_2_·2TOPO via eq 3 and the TOPO stoichiometry is confirmed by a Job plot (Figure S14). The relative Lewis acidity of isomers **(5)** and **(6)** was estimated via DFT calculations. We calculated the enthalpy change upon removal of one TOPO molecule from the structure (i.e., dissociation of the Lewis acid-base adduct). The Δ*H* was more positive for the cis structure **(5)** than for the trans structure **(6)**, thus suggesting that **(5)** is more Lewis acidic and has a higher ^31^P chemical shift (Figure S15). To further confirm the structural assignments of **(5)** and **(6)**, ^31^P chemical shifts for 6 compounds were determined from DFT calculations. The chemical shifts for the validation compounds **(1)**, **(2)**, **(4)** and TOPO were correctly ranked and the difference between computed and observed chemical shifts was between −0.7 and + 1.4 ppm compared to the absolute chemical shifts ranging from 42.0 to 79.8 ppm; see Table S1. Given that for compounds **(5)** and **(6)** the experimental difference is 4.9 ppm, the accuracy of the computations (∼2 ppm) is sufficient to assign their structures from computed ^31^P shifts. Indeed, species **(5)** has a computed δ = 66.1 ppm whereas **(6)** is shifted down to δ = 62.9 ppm consistent with the experimentally determined shifts (66.4 and 61.5 respectively). Overall, the computed chemical shifts correlate linearly (*R*^2^ = 0.996) with the experimental values (Figure S16). Further validation can be gleaned from computed ^1^H shifts at the same level of theory; see Table S2. Hence the computed chemical shifts for the DFT-optimized structures support our assignments in Figure 4.

Finally, we mixed ZrCl_4_ and Zr(O*i*Pr)_4_·*i*PrOH in the ratio 0.5:1, 1:1, 2:1, and 3:1 (with 2 TOPO equivalents), and observed the expected species in the ^31^P NMR spectrum (Figure S17)
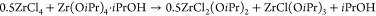
4
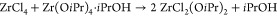
5
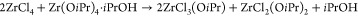
6
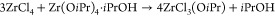
7We also confirmed the ^31^P NMR shift of ZrCl(O*i*Pr)_3_·2TOPO **(7)** as 58.7 ppm by synthesizing it according to eq 3, Figure S18. The Lewis acidity of the different zirconium species **(2)**–**(7)** decreases with every Cl to O*i*Pr substitution and this is evident by the progressive shift to lower ppm values and also by the presence of free TOPO in case of ZrCl_2_(O*i*Pr)_2_·2TOPO and ZrCl(O*i*Pr)_3_·2TOPO. For the latter two, coordination of TOPO appears an equilibrium instead of a complete reaction.

From the titration in Figure 4 and the Job plots in Figures S13, S14, and S18, it appears that, at lower than two TOPO equivalents, more Lewis acidic species are formed than expected based on the composition of the mixture. We infer that TOPO, as a strong Lewis base, causes a ligand redistribution of the precursor mixture to maximize the strength of the formed Lewis acid-base complexes. Taking for example ZrCl_2_(O*i*Pr)_2_ with 1 equiv of TOPO
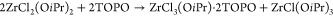
8As such, TOPO is coordinated to the strongest Lewis acid, ZrCl_3_(O*i*Pr), and only a single peak is observed in the ^31^P NMR spectrum. Even though the species ZrCl(O*i*Pr)_3_ exists in solution, it is not detected by ^31^P NMR since it is not coordinated by TOPO. To our knowledge, this is a unique and previously unreported ligand redistribution of metal complexes, induced by neutral Lewis base (L-type ligands). The only precedent for this reaction is a thermally induced ligand redistribution upon distillation of titanium chloroalkoxides.^56^ Although this was called a disproportionation in 1952, the term seems currently reserved for redox reactions.

### Evolution of Species in a Nanocrystal Synthesis

**Figure 5:**
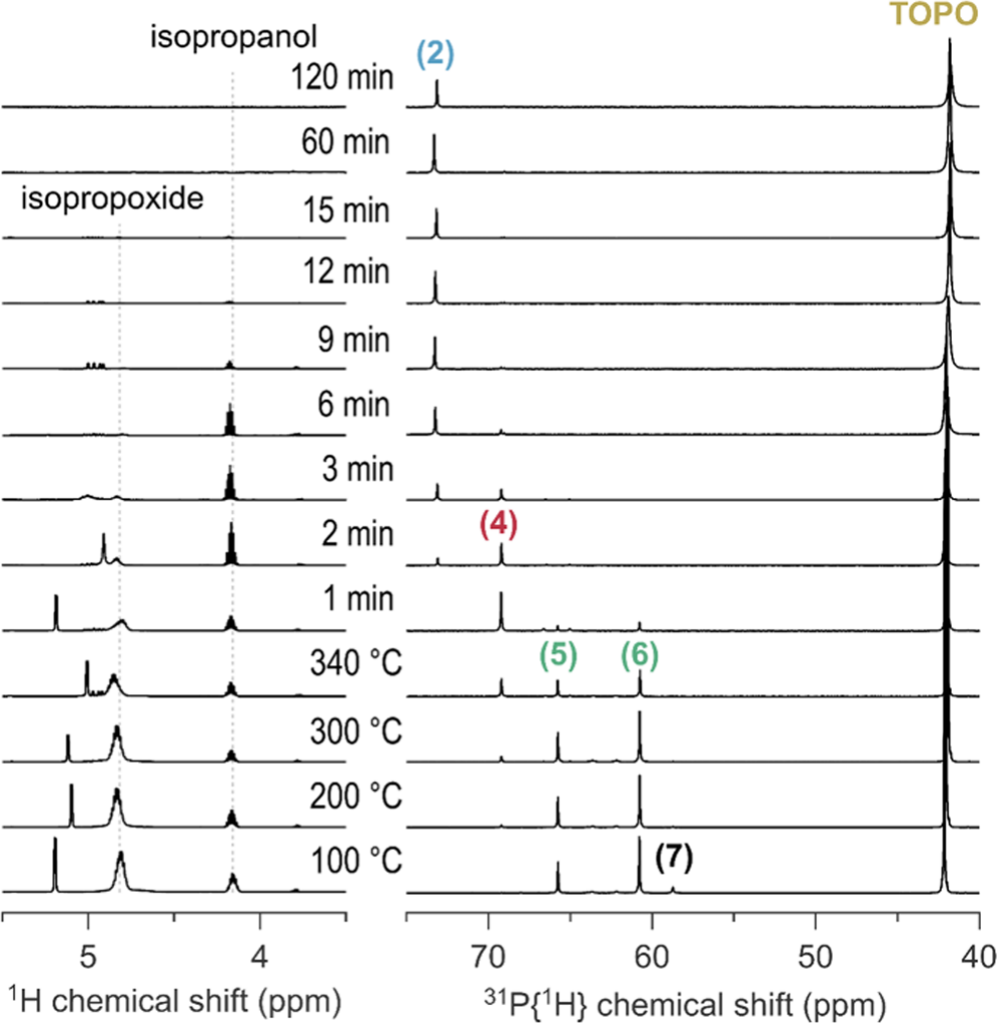
^1^H and ^31^P NMR of the reaction mixture with 1 equiv of ZrCl_4_ in C_6_D_6_. Aliquots were taken at different temperatures during the ramp and at different times at the final reaction temperature of 340 °C.

Having determined the identity of all the species in ^31^P NMR, we are now ready to investigate a real reaction (1 mmol ZrCl_4_, 1 mmol Zr(O*i*Pr)_4_·*i*PrOH and 13 mmol TOPO), see Figure 5. The zirconium molality in the reaction is 0.4 mol/kg, corresponding to Zr:TOPO = 1:6.5. We take aliquots from the reaction mixture and measure them in ^1^H and ^31^P NMR (Figure 5). At 100 °C, we find mostly species **(5)** and **(6)** in the ^31^P NMR spectrum, showing that the actual reagent in this reaction is ZrCl_2_(O*i*Pr)_2_. Upon heating to 340 °C, the isopropoxide resonance (^1^H NMR spectrum) decreases in intensity and concomitantly, **(5)** and **(6)** decrease in concentration and ZrCl_3_(O*i*Pr) **(4)** is formed. The precursor decomposition proceeds rapidly at 340 °C evidenced by the decay of the isopropoxide signal within 10 min. After 1 min at 340 °C, **(4)** is the dominant species, which then also decays within 10 min (showing thus a good correlation between the ^1^H and ^31^P NMR data). We clearly observe ZrCl_4_·2TOPO **(2)** as a by-product of the reaction. Also, signals of free isopropanol remain present in the ^1^H NMR spectrum up to 15 min despite its low boiling point. Isopropanol co-exists with **(2)**, thus showing little reactivity towards ZrCl_4_·2TOPO. This observation stands in contrast to the reactivity of isopropanol with uncoordinated ZrCl_4_ (see eq 2).

The presence of **(2)** at the end of the reaction explains why the reaction does not reach 100% yield. After isolation of the final nanocrystals and subtraction of the organic mass (determined from TGA), we reproducibly find a yield of about 60% in zirconia. This value agrees perfectly with our estimation of the yield from integrating the ^31^P NMR spectrum at the end of the reaction (Figure S19). The reaction mixture with ZrCl_4_·2THF shows qualitatively very similar trends (Figure S20), but isopropanol and THF linger in the reaction mixture to the end (2 h).

**Figure 6:**
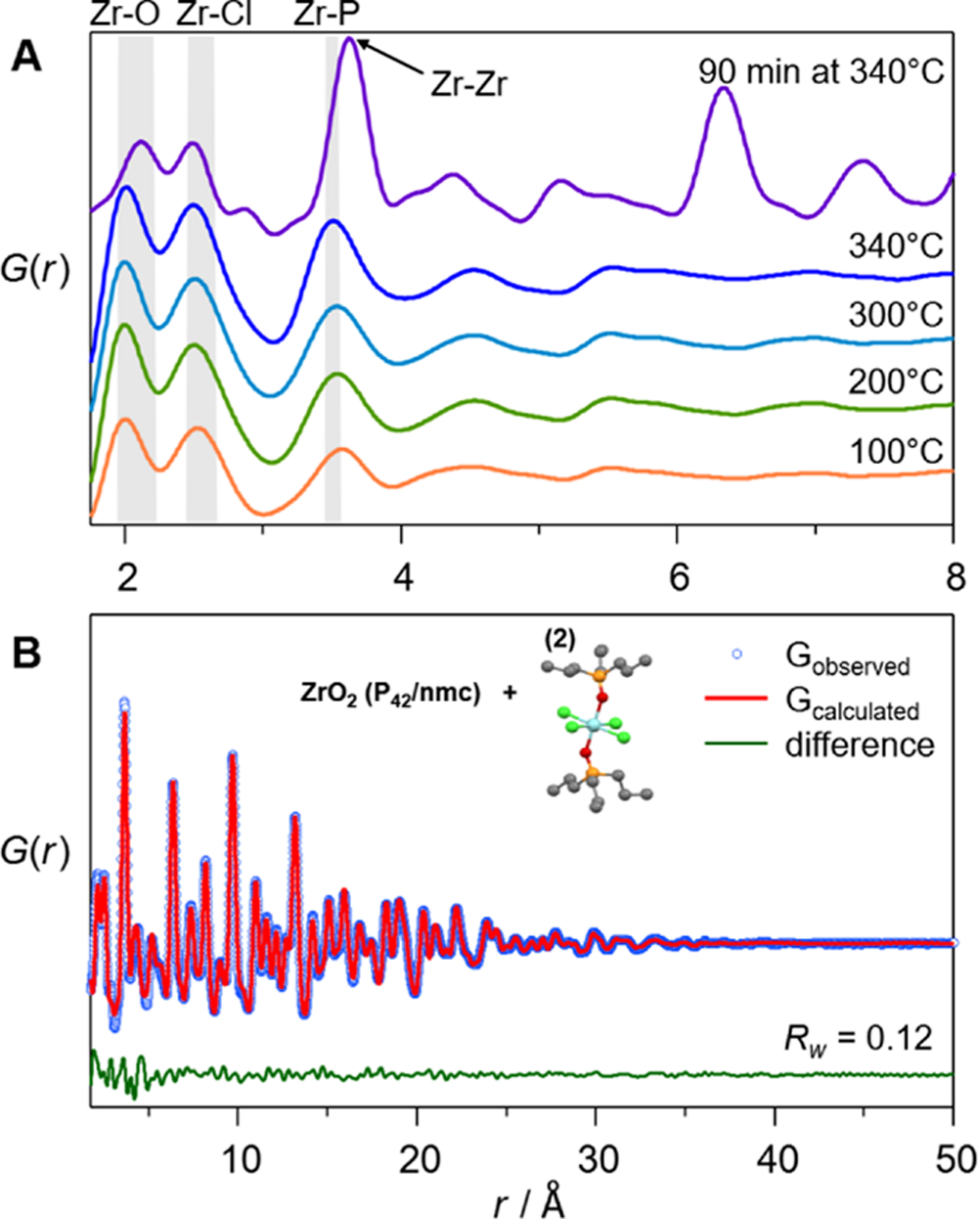
(A) X-Ray PDFs (acquired at 80 °C to melt TOPO) of reaction mixtures heated to different temperatures as indicated. The range of distances as determined from the DFT optimized structures of **(5)** and **(6)** are indicated by the grey zones. (B) PDF refinement for the reaction product at 340 °C after 90 min, using a dual-phase model with the tetragonal zirconia (*P*42/*nmc*) and the DFT optimized ZrCl_4_·2TPPO complex **(2)**. The refined values are shown in Table S3.

We further validate the results with X-ray total scattering and Pair Distribution Function (PDF) analysis. Figure 6A shows the PDF of the reaction mixture at different temperatures (100, 200, 300, and 340 °C) and after 90 min at 340 °C. The measurements were carried out *ex situ* on corresponding reaction aliquots. We assign the three main peaks in the reaction mixture to Zr-O (2.0 Å), Zr-Cl (2.5 Å), and Zr-P (3.5 Å) which are in good agreement with the respective distances in the DFT optimized structures of **(5)** and **(6)**: Zr-O (2.0 Å for O^*i*^Pr and 2.2 Å for TPPO), Zr-Cl (2.5 Å for cis and 2.6 Å for trans), and Zr-P (3.5 Å). The absence of a Zr-Zr distance at 3.6 Å in the PDF data acquired below 340 °C indicates that the precursors are indeed monomers, coordinated by TOPO. A different PDF is observed after 90 min at 340 °C, where the contribution of crystalline ZrO_2_ is prominent. The appearance of an intense Zr-Zr distance at 3.6 Å and the longer-range interactions above 5 Å indicate the formation of ZrO_2_ nanocrystals. A single phase refinement of the data using the tetragonal (*P*42/*nmc*) zirconia crystal structure results in a moderately good fit (*R*_w_ = 0.26), see Figure S21. While this crystal model describes the long-range interactions quite well, there is a considerable misfit below 5 Å. It is also clear from Figure 6A that there are still Zr-Cl distances in the sample, which are not accounted for by the model. Therefore, we performed a dual phase refinement of tetragonal zirconia and the ZrCl_4_ complex **(2)**, see Figure 6B. For the complex, we used the ZrCl_4_·2TPPO structure optimized by DFT with distances: Zr-O (2.1 Å), Zr-Cl (2.5 Å), and Zr-P (3.5 Å). The atomic positions were fixed during the PDF refinement and only the scale factor and the atomic displacement parameters are refined (Table S3). An excellent fit is obtained (*R*_w_ = 0.12), showing that the DFT structure is consistent with the PDF data and underscoring the results from NMR. The formation of ZrCl_4_ as a reaction by-product is thus firmly established.

### Quantification of Reagent Disappearance and Formation of Co-products

**Figure 7:**
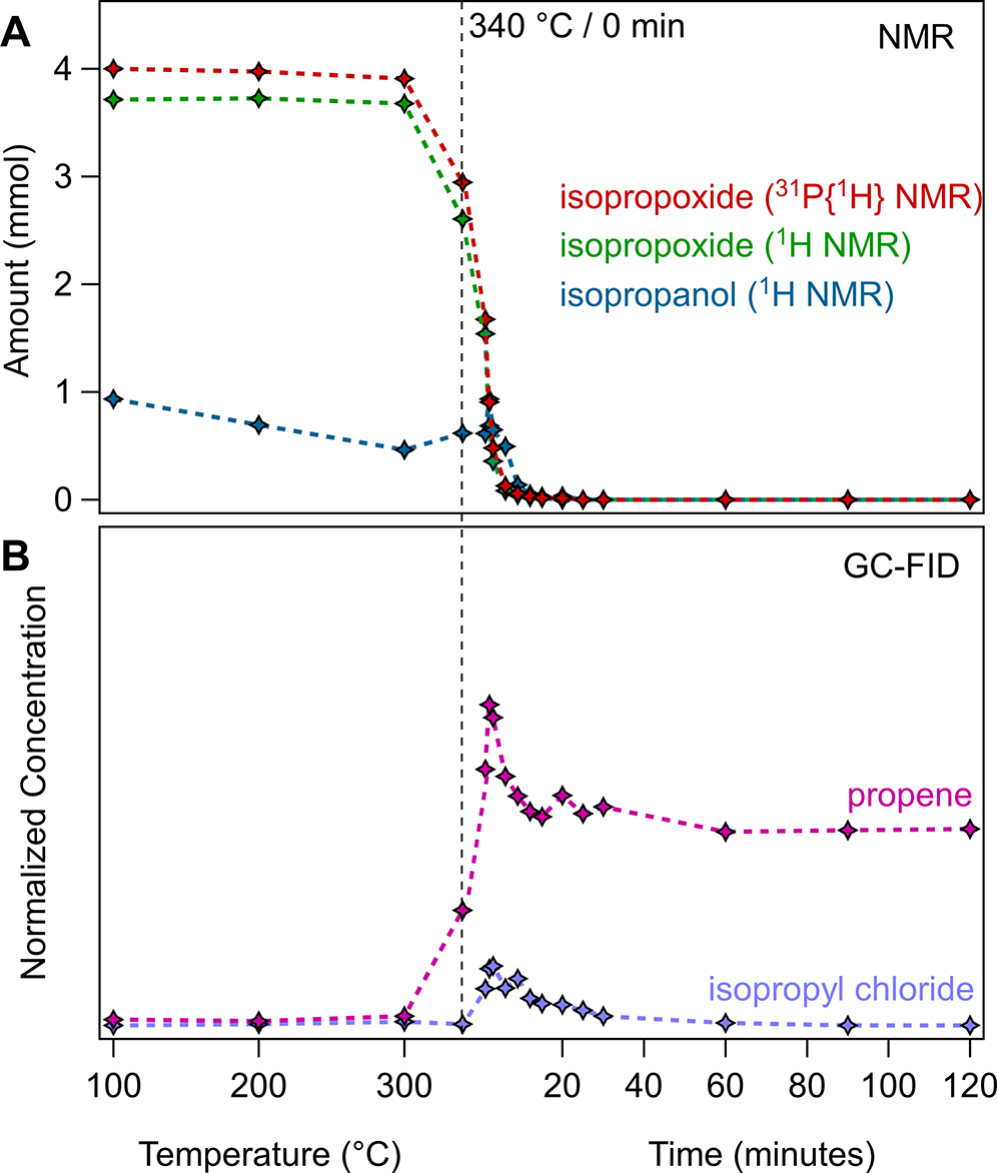
(A) Concentration of various intermediates at different times at the final reaction temperature of 340 °C for a 1:1 mixture of ZrCl_4_:Zr(O*i*Pr)_4_·*i*PrOH. The amount of different species is calculated corresponding to the integrals of TOPO bound to the Zr-centers in ^31^P NMR. (B) Normalized, relative concentration of propene and isopropyl chloride in the reaction headspace at different temperature/time points.

Based on the ^1^H NMR spectra of Figure 5 and using the methyl resonance of TOPO as the internal standard, we quantified the disappearance of isopropoxide. The same quantification was done using the ^31^P NMR spectra, taking all species **(4)**–**(7)** together and taking into account the amount of isopropoxide in every species. For both quantifications, we assumed that the total amount of TOPO remains constant during the reaction, which is a fair assumption since only a very minor fraction decomposes.^41^ Furthermore, we quantified the amount of free isopropanol in the mixture, see Figure 7A. Regarding the reaction co-products, we have already identified ZrCl_4_, but also isopropyl chloride and propene are expected based on Scheme 1. In earlier studies, isopropyl chloride and propene have been qualitatively detected.^26^ Here, we use gas chromatography with flame ionization detection (GC-FID) to compare the relative concentration of gaseous byproducts in the reaction headspace at different stages of the reaction, see Figure 7B.

From Figure 7, we learn that the isopropoxide starts decomposing between 300 and 340 °C. Interestingly, this coincides with an increase in detected isopropanol, while it was decreasing (evaporating) during the heat-up. This observation suggests that isopropanol is being released during the reaction and is thus also a co-product. Concomitant with the isopropoxide disappearance, we detect propene at 340 °C, the concentration of which reaches a maximum at 5 min at 340 °C. Isopropyl chloride is also detected but in lower concentrations. It was earlier proposed that isopropyl chloride is the actual reaction product and subsequently decomposes into HCl and propene. However, we find that propene appears before isopropyl chloride. Control experiments indicate that isopropyl chloride is indeed converted into propene, but the rate of this transformation is moderate and does not support an instantaneous conversion of isopropyl chloride (Figure S22). For this reason, we infer that propene is also the direct reaction product of the decomposition of isopropoxide. Note that pure zirconium isopropoxide also thermally decomposes around 340 °C, yielding propene and isopropanol as co-products, thus further strengthening our hypothesis.^57^

### Overall Reaction Mechanism

**Scheme 2:**
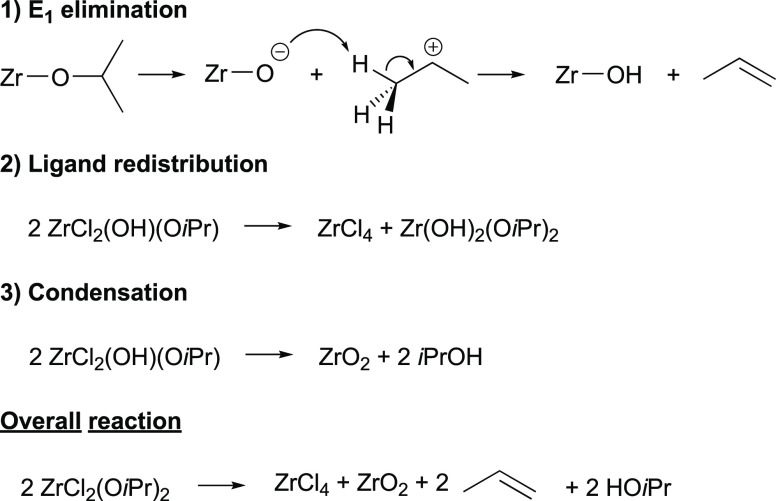
Our Alternative Pathway for the Formation of Zirconia Nanocrystals Is Based on E1 Elimination, Ligand Redistribution, and Condensation Reactions

From the above data, we conclude that the reaction mechanism is not only based on S_N_1 nucleophilic substitution (producing isopropyl chloride) but also on the E1 elimination mechanism. We propose a simplified pathway in Scheme 2, that agrees with all our observations. First, one isopropyl group in ZrCl_2_(O*i*Pr)_2_ leaves as a cation and further eliminates a proton, forming both propene and a Zr-OH moiety. Second, the zirconium complex undergoes ligand redistribution, forming both ZrCl_4_ and Zr(OH)_2_(O*i*Pr)_2_. Third, the latter condenses into ZrO_2_, eliminating two isopropanol molecules. Of course, reality will be more complex. The ligand redistribution and condensation steps most likely happen simultaneously. In addition, other condensation steps can be conceived, with the elimination of HCl or water. However, both these elimination products can react with the isopropoxide groups, releasing isopropanol and the overall sum of the reactions will be the same as the one presented in Scheme 2. Note, we do not propose that the ZrO_2_ unit is formed as the monomer in this reaction. The current reaction scheme is highlighting the formation of organic by-products and remains agnostic as to the precise crystallization mechanism of ZrO_2_. For example, the transient formation of HCl could be important to introduce the necessary reversibility in bond making and breaking that is required for crystallization. The merit of Scheme 2 lies in providing a fully balanced chemical equation for the reaction. From this chemical equation, it is self-evident that the reaction is limited to a 50% yield. The fact that the experimentally observed yield is 60% and that isopropyl chloride is also detected as a co-product, means that both the E1 elimination and the S_N_1 mechanism are active simultaneously. Indeed, for the S_N_1 mechanism, one can expect a theoretical yield of 100%
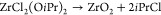
9Given that 40% of zirconium is retrieved as ZrCl_4_ at the end of the reaction, we estimate the relative contribution of the E1 and S_N_1 mechanism as 80/20.

### Size Control and Yield Optimization

**Figure 8:**
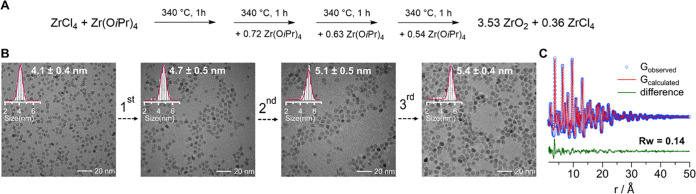
(A) Scheme showing the Zr(O*i*Pr)_4_·*i*PrOH injection strategy to increase particle size and yield. (B) TEM and histogram of particles before and after each injection. The average size is indicated. (C) PDF fit for the purified product after three injections with the tetragonal zirconia (*P*42/*nmc*) model. The refined crystallite diameter is 5.33 nm. The other refined values are shown in Table S4. The PDF fit for purified particles before injection is shown in Figure S25.

With the reaction mechanism at hand, we can introduce control over the nanocrystal size, while at the same time improving the chemical yield. We repeatedly injected extra Zr(O*i*Pr)_4_·*i*PrOH in the reaction flask, hypothesizing that it would undergo ligand redistribution with the ZrCl_4_ by-product and form the active precursor once again (Figure 8A). ^1^H and ^31^P NMR measurements of aliquots taken before and after each injection, confirm that ZrCl_4_ is converted to the mixed chloroalkoxide species upon injection of Zr(O*i*Pr)_4_·*i*PrOH (Figure S23). Each time, less ZrCl_4_ is regenerated before the next injection. By TEM analysis we observe that the ZrO_2_ nanocrystals grow from their usual 4.1 ± 0.4 nm (diameter) to 4.7 ± 0.5 nm after the first injection. By repeating the injection of extra Zr(O*i*Pr)_4_·*i*PrOH precursor, we can further increase the nanocrystal size up to 5.1 ± 0.5 and 5.4 ± 0.4 nm after the second and third injection respectively. The size dispersion of the main distribution decreases from 9.7 to 7.4% throughout this seeded growth process. However, some smaller particles (*d* = 3–4 nm) were also observed with TEM, suggesting independent nucleation of new particles and thus not a pure growth process. This is confirmed by considering the yield of the reaction. The overall yield after three injections is determined (by thermogravimetric analysis, Figure S24) to be 79%, compared to 60% for a regular synthesis. Based on the experimental yield and in the absence of nucleation, one would have expected the particles to have grown to 5.8 nm, instead of the observed 5.4 nm. Therefore, we conclude that nucleation and growth take place simultaneously. There is a difference between the experimental yield and the yield based on the reaction scheme in Figure 8A (91%), which could be due to loss of smaller particles during purification, unreacted monomer at the end of the reaction, or a slight error on the final ZrCl_4_ amount. Finally, the crystalline phase and size of the final product were verified by X-ray PDF (Figure 8C). The refinement using the tetragonal zirconia structure yields a crystallite size of 5.33 nm, which is very close to the size obtained from TEM, and thus confirms the monocrystalline nature (and epitaxial growth) of the nanocrystals.

### Generality of the Mechanism

To generalize our findings to other metal oxide nanocrystal systems, HfO_2_ and TiO_2_ nanocrystals are synthesized in TOPO. Slightly different reaction temperatures are used for HfO_2_ (360 °C) and TiO_2_ (300 °C) depending on the reports in the literature.^27,58^ For hafnia, the ^31^P NMR spectra of the reaction aliquots (Figure S26) confirm the formation of similar active precursors (HfCl_2_(O*i*Pr)_2_) and the co-product HfCl_4_·2TOPO. The ^31^P NMR shift of HfCl_4_·2TOPO is independently verified by a control experiment (Figure S27). Furthermore, upon repeated injection of additional Hf(O*i*Pr)_4_·*i*PrOH, the nanorods grow in length from 14.7 to 24.9 nm (Figure S28), as also shown previously.^36^ The overall yield of the synthesis increases from 30 to 63% (see TGA analysis in Figure S28). For titania, we found neither conclusive evidence for the mixed chloroalkoxide species, nor for a TiCl_4_ by-product (Figure S29). We conclude that the formation of HfO_2_ follows the same synthetic pathway as for ZrO_2_ while the case of TiO_2_ requires further research. The differences between titania on the one hand and zirconia and hafnia on the other hand, are most likely responsible for the difficulties in preparing solid solutions of titania with zirconia or hafnia.^30^ Finally, ZrBr_4_ is also often used as precursor instead of ZrCl_4_ and the resulting nanocrystals are smaller (*d* = 3 nm).^26^ It is tempting to assume the reaction follows a similar reaction mechanism as described above. While we also find ligand redistribution effects for a 50/50 mixture of ZrBr_4_ and Zr(iOPr)_4_·*i*PrOH (with a varying amount of TOPO added), the situation looks more complex and identifying the exact species requires further research (Figure S30).

## Conclusions

We elucidated the precursor chemistry in the synthesis of zirconia and hafnia nanocrystals from metal chloride and metal isopropoxide in TOPO. We showed how TOPO coordinates to the different precursors and that the mixed chloroalkoxide is the actual precursor in the reaction. Interestingly, we found a ligand redistribution reaction that is controlled by the amount of added neutral Lewis base (L-type). By supplying a sub-stoichiometric amount of Lewis base, the system redistributes the (X-type) ligands to create more Lewis acidic species and thus maximizes the strength of the Lewis acid-base adduct. We also monitored how the reaction mixture decomposes at 340 °C and established the formation of ZrCl_4_ and isopropanol as by-products. The combination of NMR spectroscopy, DFT calculations at the B3LYP/aug-cc-pVDZ level of theory, and X-ray PDF analysis provided a comprehensive and consistent structural and molecularly refined characterization of precursors and intermediates/by-products. We further quantified the other gaseous by-products by quantitative GC and found that propene is the dominant by-product, with also isopropyl chloride being detected. These results lead us to hypothesize an alternative precursor decomposition mechanism which is based on E1 elimination of propene, ligand redistribution to form MCl_4_ and M(OH)_2_(O*i*Pr)_2_, and finally condensation to MO_2_ with the formation of isopropanol. Based on the yield we estimate the ratio of the S_N_1 and E1 mechanism to be 20–80 for ZrO_2_. Finally, we used the MCl_4_ by-product as an opportunity to control the nanocrystal size. Using a seeded growth approach, we periodically injected metal isopropoxide. The latter forms again the active precursor after reaction with MCl_4_, and the nanocrystals grow further. We thus introduced a valid pathway to gain control over nanocrystal size, which is particularly challenging for group 4 and 5 metal oxides. In addition, the fundamental insights obtained above will enable the formation of even more complex oxide nanocrystals, which will serve as valuable building blocks in material science.

## Experimental Section

### Materials

ZrCl_4_ (99.9%), HfCl_4_ (99.9%) and Ti(O*i*Pr)_4_ (98%) were purchased from Strem Chemicals. TiCl_4_ (99.9%) was bought from ACROS Organics. Zr(O*i*Pr)_4_·*i*PrOH (99.9%), Hf(O*i*Pr)_4_·*i*PrOH (99.9%), toluene (99.5%) and, acetone (99.8%) were purchased from Sigma-Aldrich and used without further purification. Deuteroform (99.8 atom %) was purchased from Cambridge Isotope laboratories and Benzene-D6 (99.5 atom %) from Apollo scientific, 10/100 mL of activated 4 Å molecular sieves were added and left to stand for 3 days in the glovebox to remove residual water. 3 mm high-throughput NMR tubes (0.58 mm wall thickness) were purchased from Sigma-Aldrich. Tri-*n*-octylphosphine oxide (99%) was bought from Strem chemicals and recrystallized according to Owen et al.^59^ ZrCl_4_·(THF)_2_ was synthesized according to Manzer et al.^60^

### General Instrumentation

Nuclear Magnetic Resonance (NMR) measurements were recorded at 298K on Bruker UltraShield 500 spectrometer operating at a frequency of 500.13 MHz. ^31^P spectra were acquired using inverse gated decoupling, a delay time of 1 s and 64 scans. The ^31^P spectra were processed with a line broadening of 5 Hz. Spectra acquired with a delay of 5 s gave identical relative integrations. Transmission electron microscopy (TEM) images (of a drop-cast suspension on a Holey Carbon Film–Cu grid) were taken on FEI Talos F200C TEM with 200 kV FEG optics.

### Nanocrystal Synthesis

Zirconia nanocrystals are synthesized according to a previously published procedure that involves mixing the reagents at room temperature and heating the mixture up to 340 °C.^41^ Typical amounts were: 10 g recrystallized TOPO, Zr(O*i*Pr)_4_·*i*PrOH (0.775 g, 2 mmol), and ZrCl_4_·2THF (0.754 g, 2 mmol). A synthetic variation uses ZrCl_4_ (0.466 g, 2 mmol) instead of ZrCl_4_·2THF. For enhancing the reaction yield, Zr(O*i*Pr)_4_·*i*PrOH was dissolved in TOPO (6.5 mmol TOPO / 1 mmol Zr), heated to 100 °C and rapidly injected into the reaction mixture at 340 °C. The temperature of the mixture decreases to 320 °C upon injection but rapidly recovers to 340 °C. Titania and hafnia nanocrystals were synthesized using a similar heat-up method with an equimolar mixture of metal chloride and metal isopropoxide but slightly different reaction temperatures were used for HfO_2_ (360 °C) and TiO_2_ (300 °C). Nanocrystal purification is performed using acetone and toluene as non-solvent and solvent, respectively, in the quantities like previously described.^41^ To determine the yield, the dried particles are weighed and their organic fraction is determined by TGA.

### Synchrotron X-ray Total Scattering Experiments

Samples were prepared by the temporal sampling of reaction aliquot into 3 mm NMR tubes and sealed under argon atmosphere. We also used 2 mm glass capillaries from Hilgenberg for sampling, but they were prone to breaking and the data quality was worse. Data from the samples were measured at beamline P21.1 at DESY in Hamburg, Germany, and beamline ID15 at ESRF in Grenoble, France. At ESRF, X-ray total scattering data were collected at 80 °C (using a nitrogen cryo stream), in rapid acquisition mode, using a 2D Pilatus CdTe 2 M detector (172 × 172 μm^2^ pixel size) with a sample-to-detector distance of 264 mm. The incident wavelength of the X-rays was λ = 0.1441 Å (66.05 keV). Calibration of the experimental setup was performed using a silicon standard sample. At DESY, X-ray total scattering data were collected at 80 °C in a home-built aluminium heating block in rapid acquisition mode, using a 2D Varex 4343RF amorphous silicon detector (2880 × 2880 pixels and 150 × 150 μm^2^ pixel size) with a sample-to-detector distance of 800 mm. During the measurement, the sample stage was placed in a Helium filled chamber to avoid air scattering. The incident wavelength of the X-rays was λ = 0.1220 Å (101.62 keV). Calibration of the experimental setup was performed using a Ni standard.

### Analysis of Synchrotron X-ray Total Scattering Data

Raw 2D data were corrected for geometrical effects and polarization, then azimuthally integrated to produce 1D scattering intensities versus the magnitude of the momentum transfer *Q* (where *Q* = 4π sin θ/λ for elastic scattering) using pyFAI and xpdtools.^61,62^ The program xPDFsuite with PDFgetX3 was used to perform the background subtraction, further corrections, and normalization to obtain the reduced total scattering structure function *F*(*Q*), and Fourier transformation to obtain the pair distribution function (PDF), *G*(*r*).^63,64^ For data reduction, the following parameters were used after proper background subtraction: *Q*_min_ = 0.8 Å^–1^, *Q*_max_ = 16 Å^–1^, Rpoly = 0.9 Å. Modeling was carried out using Diffpy-CMI.^65^

### Lab Source X-ray Total Scattering Experiments

Experiments were conducted using a Malvern Panalytical Empyrean Nano Edition lab source PDF diffractometer with Ag Kα (0.56 Å and 22.1 keV) source. Purified samples were prepared in a 0.2 mm glass capillary. Data collection was carried out with 1D focusing X-ray mirror/slit system and a Galipix3D hybrid pixel detector attached to an 85 mm radius reduction interface using Data collector software. Data reduction was carried out in Highscore Plus with *Q*_min_ = 0.4 Å^–1^, *Q*_max_ = 20 Å^–1^.^66^ Modeling and fitting was carried out using Diffpy-CMI.^65^

### Gas Chromatography

Samples (50 μL) of the gas phase were taken from the reaction flask with a gastight syringe and injected into headspace crimp vials (10 mL) filled with nitrogen. The samples were further diluted by transferring 200 μL into a second headspace crimp vial (10 mL). They were analyzed on a gas chromatograph (SRI 8610C, SRI instruments) equipped with a Haysep D column (3 m 2 mm ID Mesh 80/100) and an FID detector. As a carrier gas N_2_ was used with a flow rate of 1 mL/min. The samples (1 mL) were injected with an autosampler (HT2000H, HTA instruments). The separation of the products was achieved with a temperate gradient starting from 70 °C (held for 2 min) and then heating to 270 °C at a rate of 10 °C/min. As a reference isopropyl chloride (99%, Sigma-Aldrich) and propene (Pangas) was used.

### Quantum Chemical Calculations

All calculations were performed with the B3LYP functional together with the aug-cc-pVDZ basis set for C, H, O, Cl, and P atoms using Gaussian09.^67,68^^69,70^ The aug-cc-pVDZ pseudopotential and associated basis set of Peterson et al. was taken from the Basis Set Exchange and applied to the Zr atoms.^71,72^ To validate the calculations at the B3LYP/aug-cc-pVDZ level of theory which yielded Δ*H* = −20.7 kJ/mol for the exchange reaction of a single THF molecule in ZrCl_4_·2THF for triethylphosphine oxide (only cis isomers considered), the enthalpies were recomputed at the M06/aug-cc-pVDZ and MP2/aug-cc-pVDZ levels of theory for which Δ*H* = −16.1 kJ/mol and Δ*H* = −20.4 kJ/mol were obtained, respectively. Thus, the formation energies are all within 4 kJ/mol (∼1 kcal/mol) which is the “chemical accuracy” expected from such calculations. As the species under investigation are already computationally demanding as per their size, calculations with several explicit solvent molecules in their optimized structures are too time consuming. Alternatively, using implicit solvent models as a substitute was deemed not sufficiently accurate given that some of the energy differences encountered are small. Therefore, only calculations in the gas phase were carried out. To calculate the ^1^H and ^31^P NMR chemical shifts from the optimized structures, we followed the protocol of Willoughby et al.^73^ The scaling factor for the ^1^H NMR chemical shifts at our level of theory was reported by Pierens.^74^

### Thermogravimetric Analysis (TGA)

All experiments were performed on a TGA5500 (TA instruments) instrument. Sample was heated to 800 °C at a rate of 5 °C/min. After maintaining the temperature for 15 min, the sample was cooled down to room temperature.

## Supporting Information Available

^1^H and ^31^P NMR spectra of titrations, aliquots and control experiments, job plots of the different complexes, PDF refinements, TGA analysis and GC-FID results (PDF)
