# Correction to “Mechanistic Insight into the Precursor Chemistry of ZrO_2_ and HfO_2_ Nanocrystals, toward Size-Tunable Syntheses”

**DOI:** 10.1021/jacsau.2c00231

**Published:** 2022-04-28

**Authors:** Rohan Pokratath, Dietger Van den Eynden, Susan Rudd Cooper, Jette Katja Mathiesen, Valérie Waser, Mike Devereux, Simon J. L. Billinge, Markus Meuwly, Kirsten M. Ø. Jensen, Jonathan De Roo

Scheme 2 in the original paper contains a mistake in the third step: condensation. The species at the left side of the arrow—2 ZrCl_2_(OH)(O*i*Pr)—is incorrect and should be Zr(OH)_2_(O*i*Pr)_2_. As a consequence, the equation is unbalanced. This error has been corrected here in Scheme 2, resulting in a balanced chemical equation where the sum of steps 1–3 results in the overall reaction equation. The text in the original manuscript was correct and all reported results remain unchanged.

**Scheme 2:**
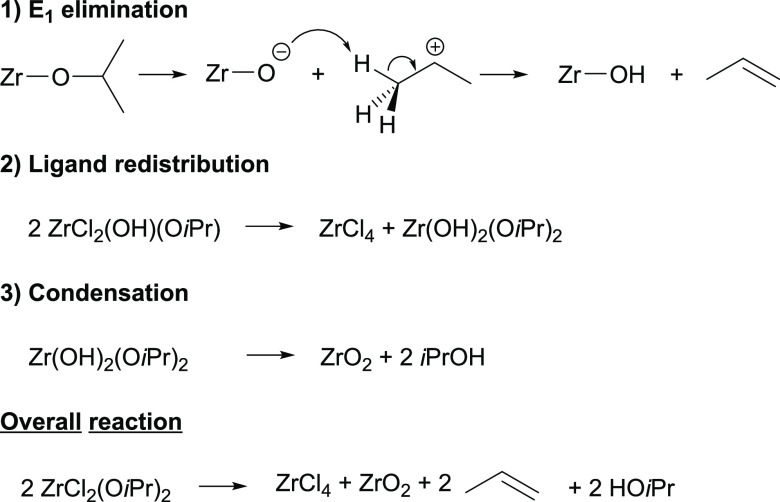
Corrected Scheme 2: Our Alternative Pathway for the Formation of Zirconia Nanocrystals Is Based on E1 Elimination, Ligand Redistribution, and Condensation Reactions

