# Supplementary material for: Mechanistic Insight into the Precursor Chemistry of ZrO2 and HfO2 Nanocrystals; towards Size-Tunable Syntheses
Source: JACS Au. 2022 Mar 9;2(4):827–38. doi: 10.1021/jacsau.1c00568 (PMC9088301; doi:10.1021/jacsau.1c00568)
Supplement: Supplementary file 1 — au1c00568_si_001.pdf [file au1c00568_si_001.pdf]

Supporting information

for

Mechanistic Insight into the Precursor Chemistry of ZrO_2 and HfO_2 Nanocrystals; towards Size-Tunable Syntheses

Rohan Pokratath,[1] Dietger Van den Eynden,[1] Susan Rudd Cooper,[2] Jette Katja Mathiesen,[2] Valérie Waser,[1] Mike Devereux,[3] Simon J. L. Billinge,[4,5] Markus Meuwly,[3] Kirsten M. Ø. Jensen,[2] Jonathan De Roo[1]*

[1] Department of Chemistry, University of Basel, Mattenstrasse 24, BPR 1096, Basel 4058, Switzerland

[2] Department of Chemistry, University of Copenhagen, Universitetsparken 5, Copenhagen Ø 2100, Denmark

[3] Department of Chemistry, University of Basel, Klingelbergstrasse 80, Basel 4056, Switzerland

[4] Applied Physics and Applied Mathematics Department, Columbia University, New York, NY 10027, USA

[5] Condensed Matter Physics and Material Science Department, Brookhaven National Laboratory, Upton, NY 11973, USA

Corresponding author: Jonathan De Roo, Jonathan.DeRoo@unibas.ch

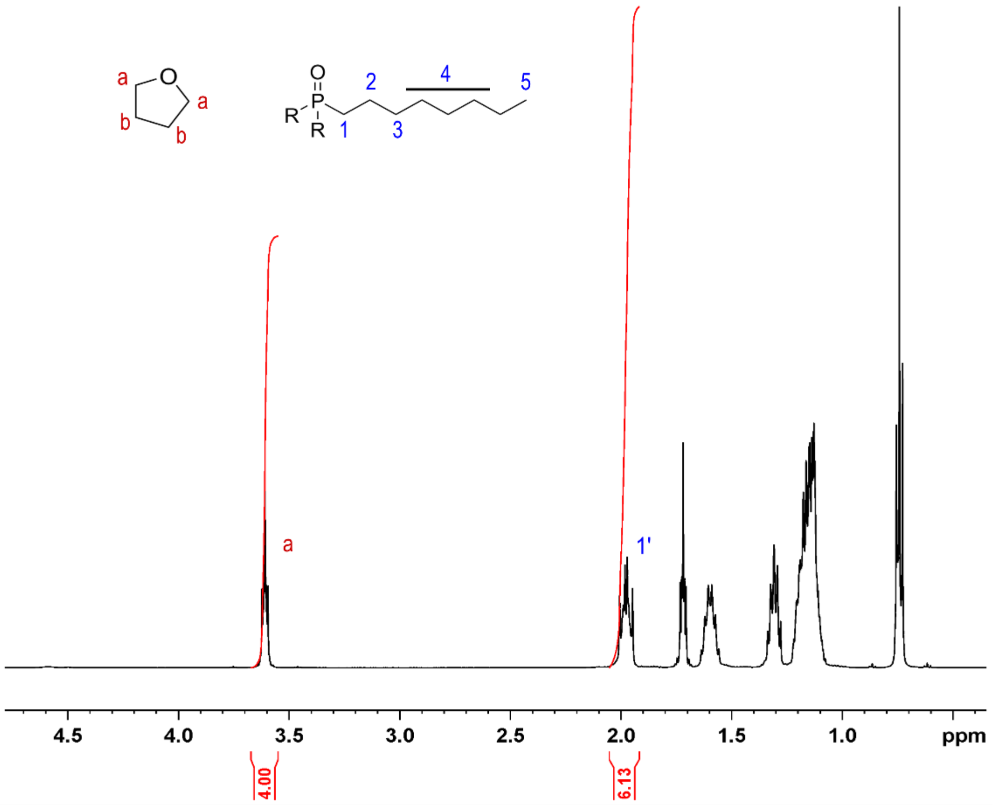

Figure S1. ^{1}H NMR in $CDCl_3$ showing the ratio of integrals for free THF and coordinated TOPO obtained by mixing $ZrCl_4$.2THF with 2 equivalents of TOPO.

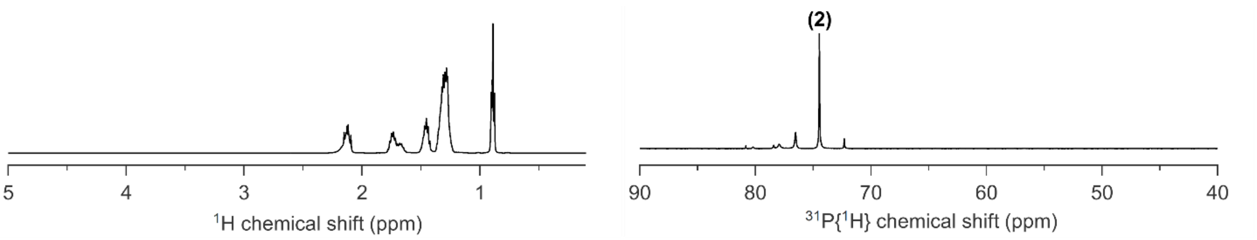

Figure S2. ^{1}H NMR and ^{31}P NMR of $ZrCl_4$ dissolved in $CDCl_3$ with two equivalents of TOPO.

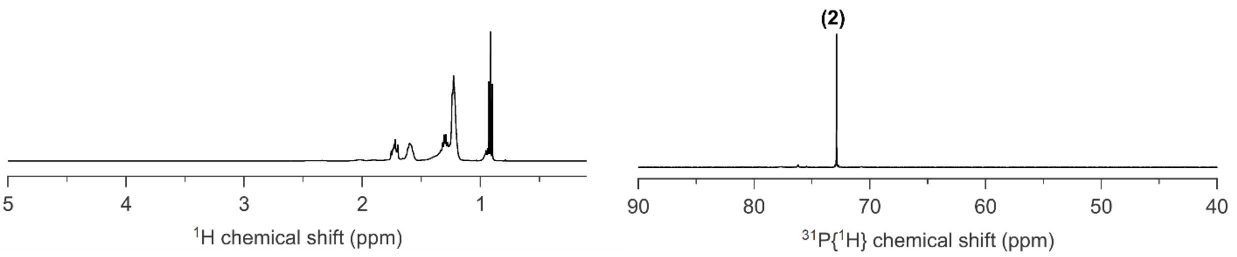

Figure S3. ^{1}H NMR and ^{31}P NMR of $ZrCl_4$ dissolved in C_6D_6 with two equivalents of TOPO.

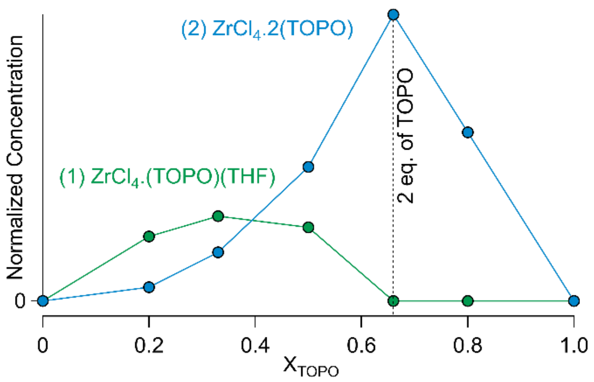

Figure S4. Job plot analysis for the binding event between $ZrCl_4.2THF$ and TOPO. The total amount of species mixed for each data point is 0.15 mmol. The asymmetry arises because of the intermediate (1) at low TOPO mole fraction.

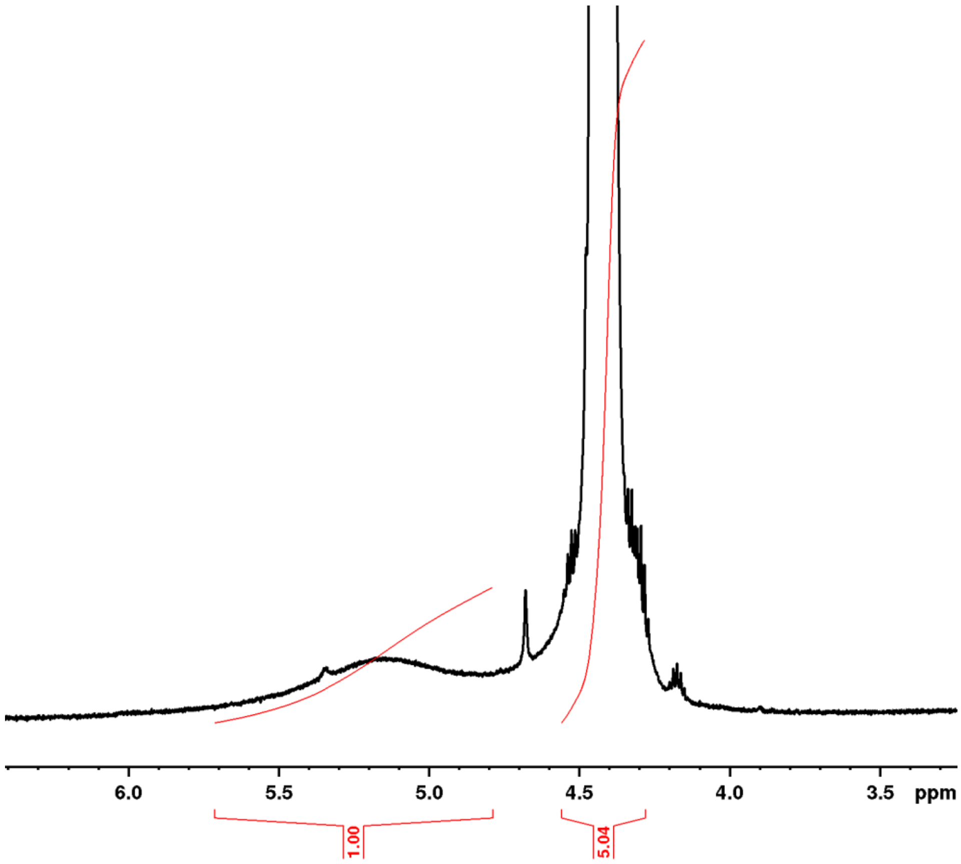

Figure S5. ^{1}H NMR spectrum of zirconium isopropoxide isopropanol complex in $CDCl_3$.

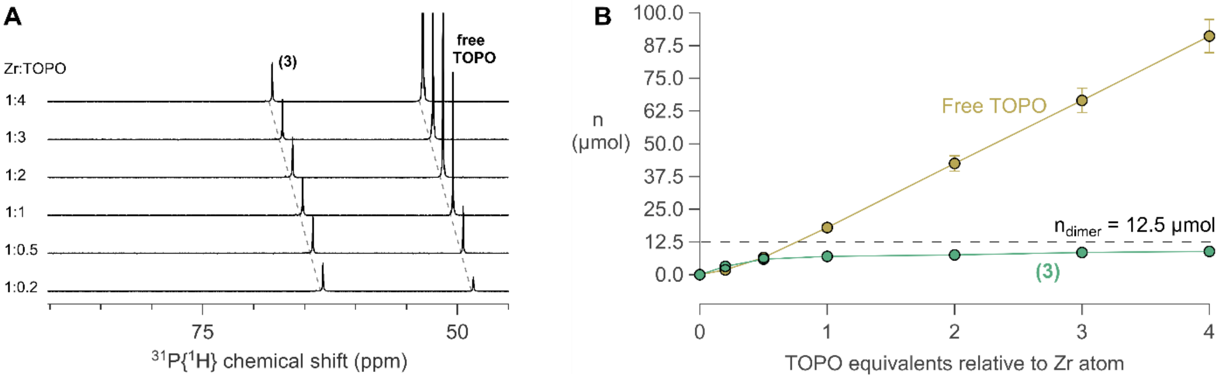

Figure S6. (A) ^{31}P NMR of the titration of a solution of 0.05M Zr(O*i*Pr)$_4$.*i*PrOH in CDCl$_3$ with equivalents of TOPO relative to Zr. The spectra have a relative x-offset of 1 ppm to each other. (B) Variation in TOPO species over the course of the titration. The total amount of Zr dimer in the sample was 12.5 µmol.

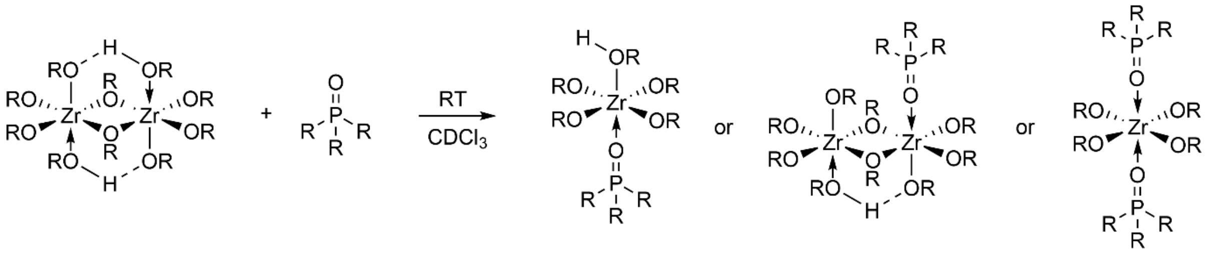

Figure S7. Scheme for the reaction of Zr(O*i*Pr)$_4$.*i*PrOH with TOPO with three possible products.

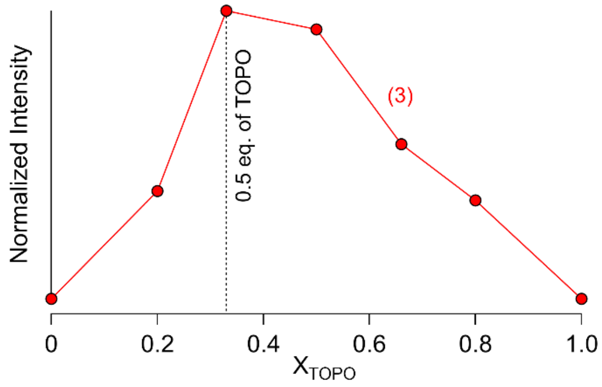

Figure S8. Job plot analysis for the binding event between Zr(O*i*Pr)$_4$.*i*PrOH and TOPO. The total amount of species mixed for each data point is 0.15 mmol.

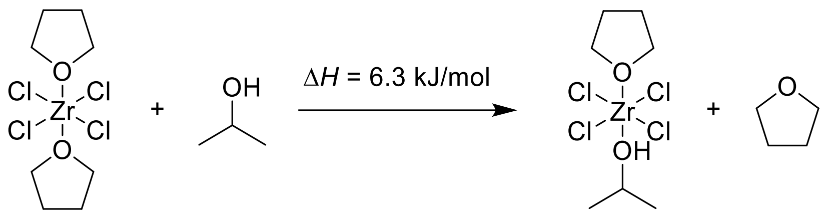

Figure S9. Δ*H* of the exchange reactions for $ZrCl_4$.2THF with *i*PrOH.

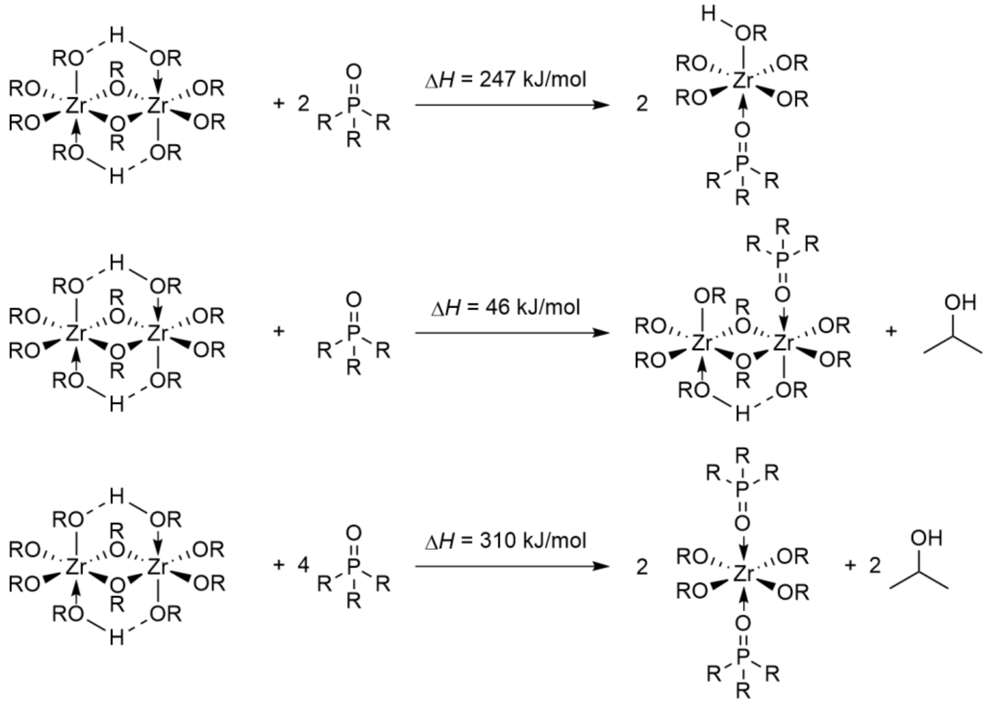

Figure S10. Δ*H* of the exchange reactions for Zr(O*i*Pr)$_4$.*i*PrOH with TPPO. (R = propyl).

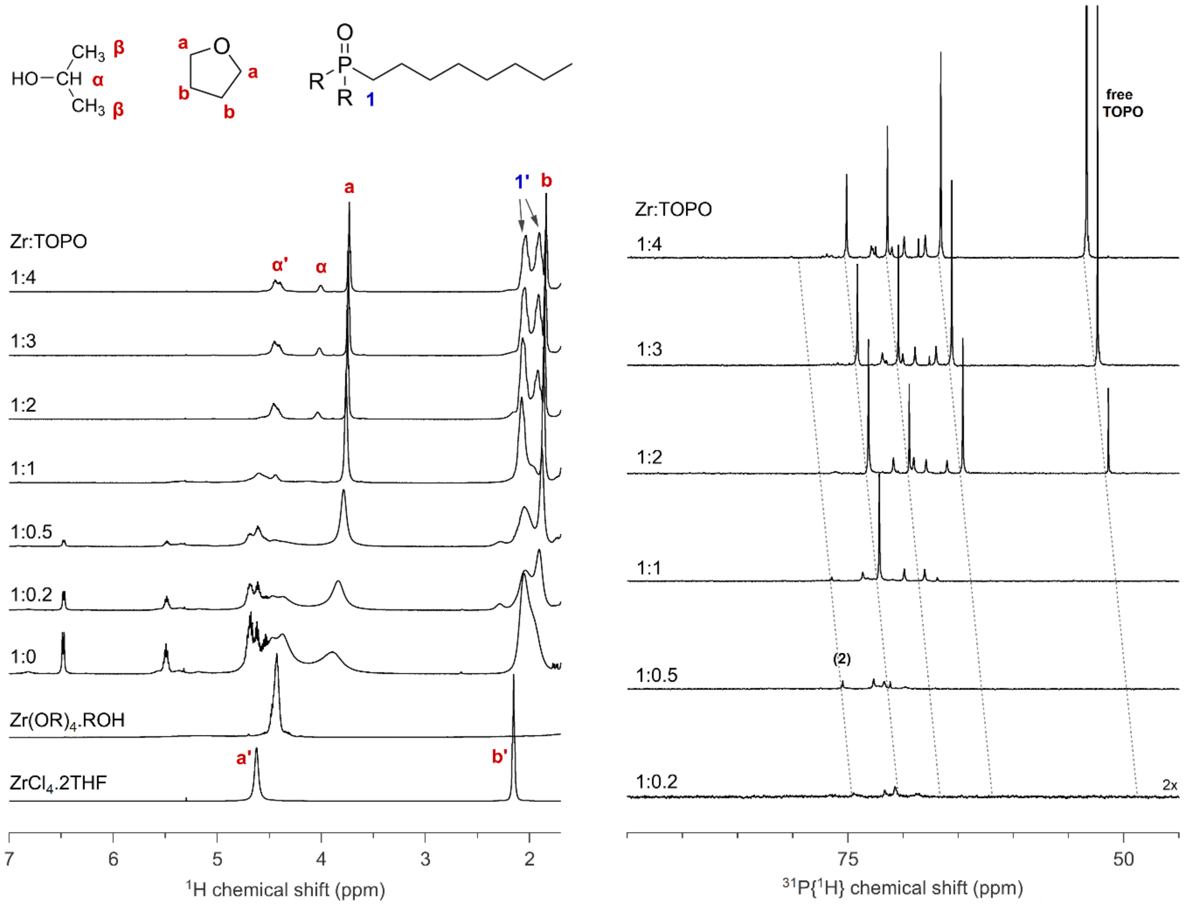

Figure S11. ^{1}H and ^{31}P NMR for the titration of a **1.25 : 1 mixture** of $ZrCl_4$.2THF : Zr(O*i*Pr)$_4$.*i*PrOH with TOPO in $CDCl_3$ at room temperature. The ratio of TOPO to Zr is indicated in the figure.

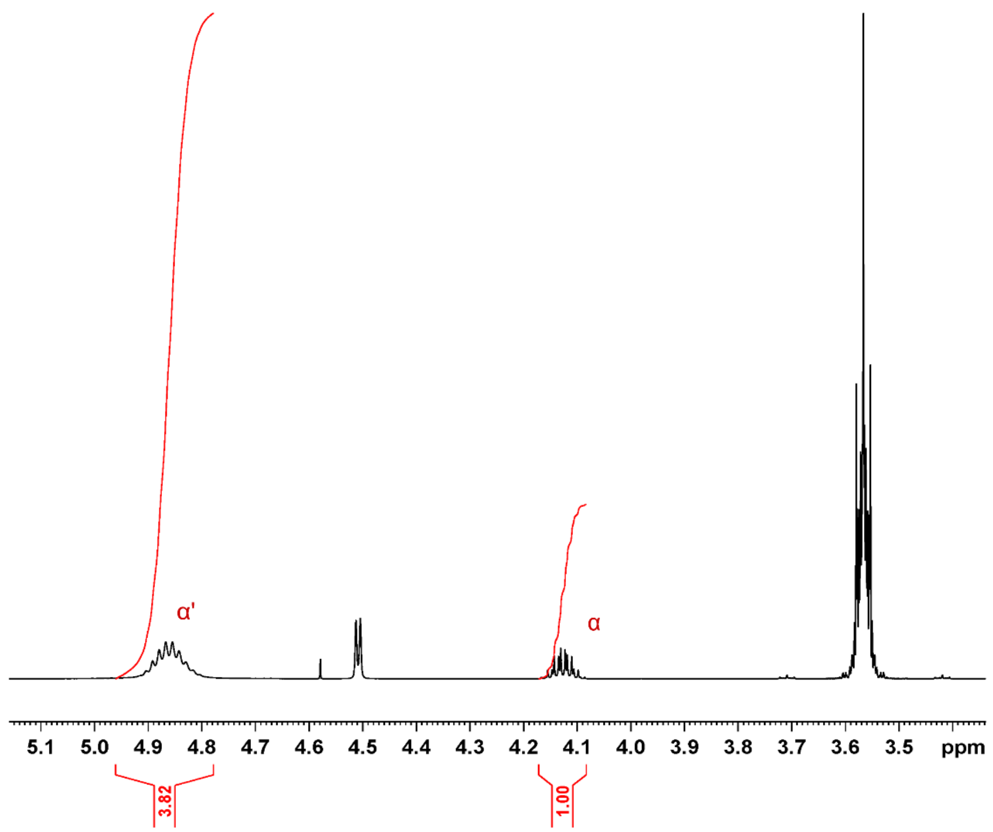

Figure S12. ^{1}H NMR showing the integral ratio between free and coordinated isopropanol for 1.25 : 1 mixture of $ZrCl_4$.2THF : Zr(O*i*Pr)$_4$.*i*PrOH with 3 eq. of TOPO in C_6D_6.

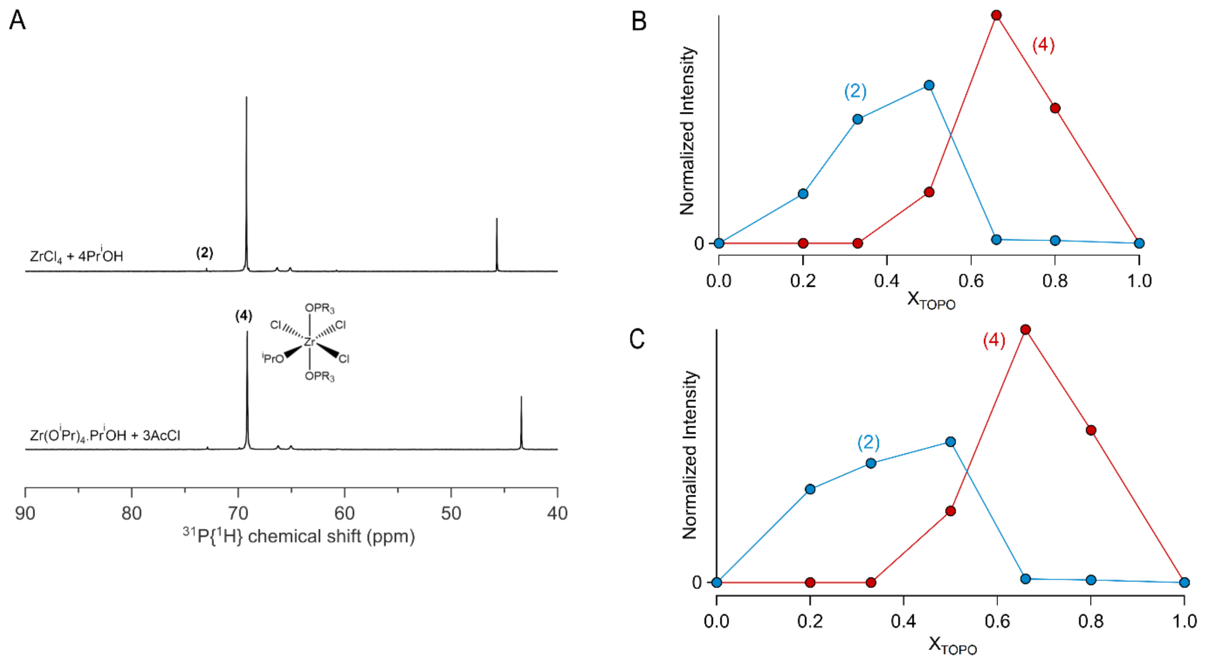

Figure S13. (A) ^{31}P NMR in C_6D_6 for the corresponding reaction product mixed with two TOPO equivalents. The structures of identified intermediates are included next to their respective peaks. The total quantity of Zr in each sample is 100 μmol. Job plot describing the binding event between the product of reaction (B) Zr(O*i*Pr)$_4$.*i*PrOH + 3AcCl, and (C) $ZrCl_4$ + 4*i*PrOH and TOPO.

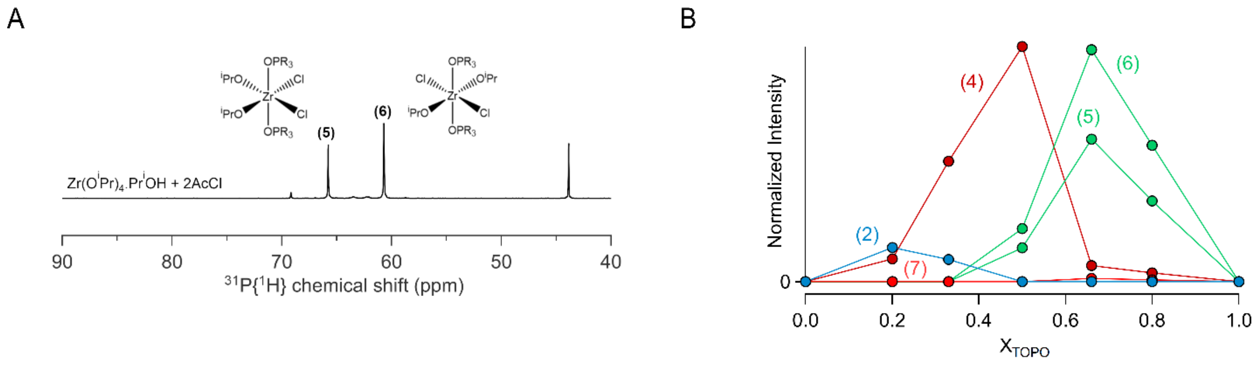

Figure S14. (A) ^{31}P NMR in C_6D_6 for the corresponding reaction product mixed with two TOPO equivalents. The structures of identified intermediates are included next to their respective peaks. The total quantity of Zr in each sample is 100 μmol. (B) Job plot describing the binding event between the product of reaction Zr(O*i*Pr)$_4$.*i*PrOH + 2AcCl and TOPO.

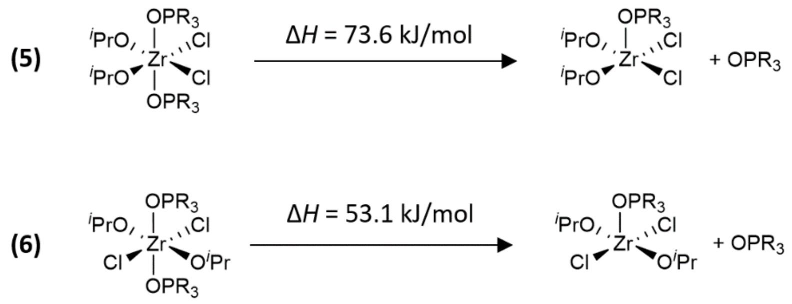

Figure S15. Δ*H* after removing one TEPO molecule from species **(5)** and **(6)** (R=ethyl chain).

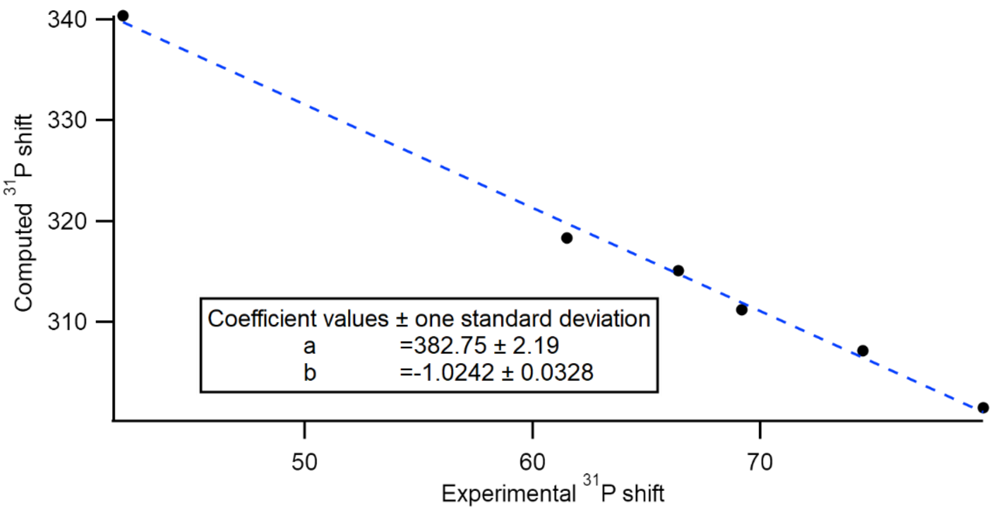

Figure S16. The unscaled, computed ^{31}P NMR chemical shifts from the optimized structures at the DFT level of theory (B3LYP with aug-cc-pVDZ) shows a good linear correlation ($R^2 = 0.996$) with the experimental chemical shifts in $CDCl_3$. From the linear fit, we extracted an *ad hoc* scaling factor and rescaled the chemical shifts, see Table S1. Please note that this scaling factor is specific for our data set since we used triethylphosphine oxide in our computations and the experimental values belong to the species with trioctylphopshine oxide. The proton chemical shifts (scaled according to the reported scaling factor in Journal of Computational Chemistry **2014**, 35 (18), 1388-1394) show also good agreement with experimental values (Table S2).

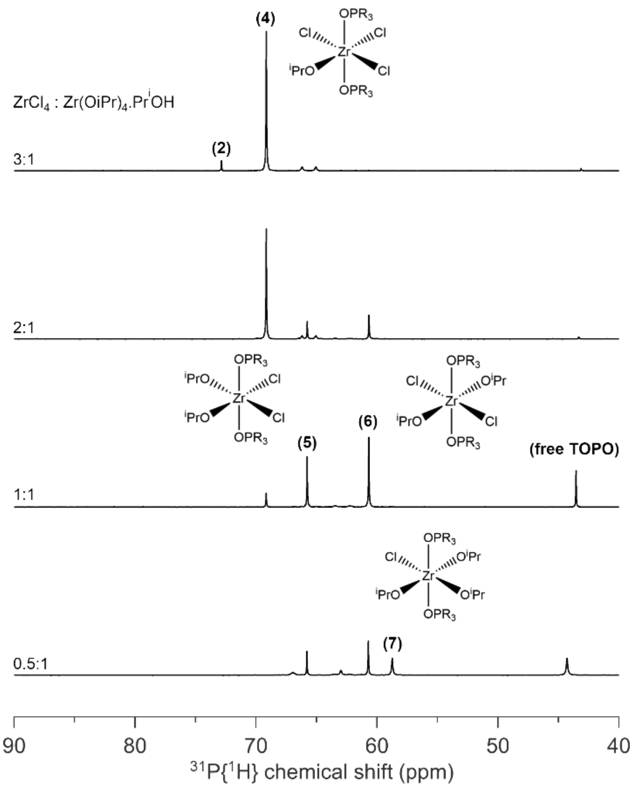

Figure S17. ^{31}P NMR of $ZrCl_4$: $Zr(O\textit{i}Pr)_4$.*i*PrOH mixture at room temperature in different ratios with two equivalents of TOPO in C_6D_6.

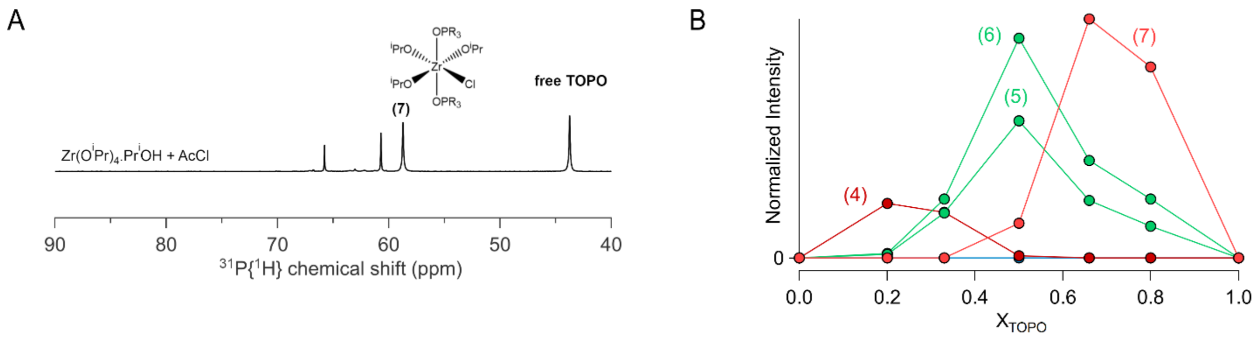

Figure S18. (A) ^{31}P NMR in C_6D_6 for the corresponding reaction product mixed with two TOPO equivalents. The structures of identified intermediates are included next to their respective peaks. The total quantity of Zr in each sample is 100 µmol. (B) Job plot describing the binding event between the product of reaction $Zr(O\textit{i}Pr)_4$.*i*PrOH + AcCl and TOPO.

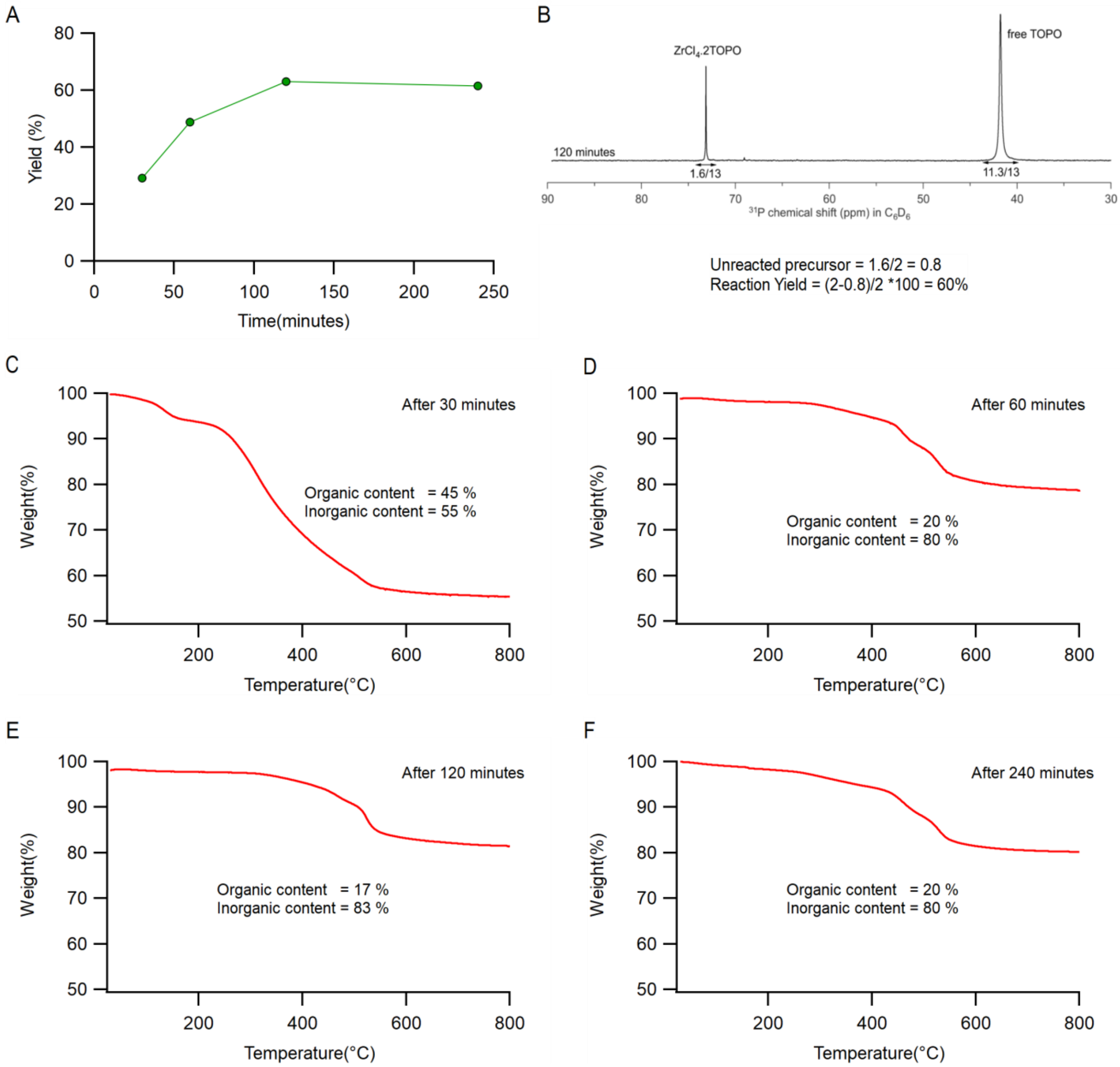

Figure S19. (A) The chemical yield of the reaction was measured by weighing the purified nanocrystals (synthesized from ($ZrCl_4$+Zr(O*i*Pr)$_4$.*i*PrOH in TOPO) and performing a TGA experiment to determine the inorganic fraction. (B) Calculation of yield with ^{31}P NMR at the end of the reaction. The TGA analysis of the purified sample after (C) 30 minutes (D) 60 minutes (E) 120 minutes and (F) 240 minutes. The determined organic and inorganic fraction are shown.

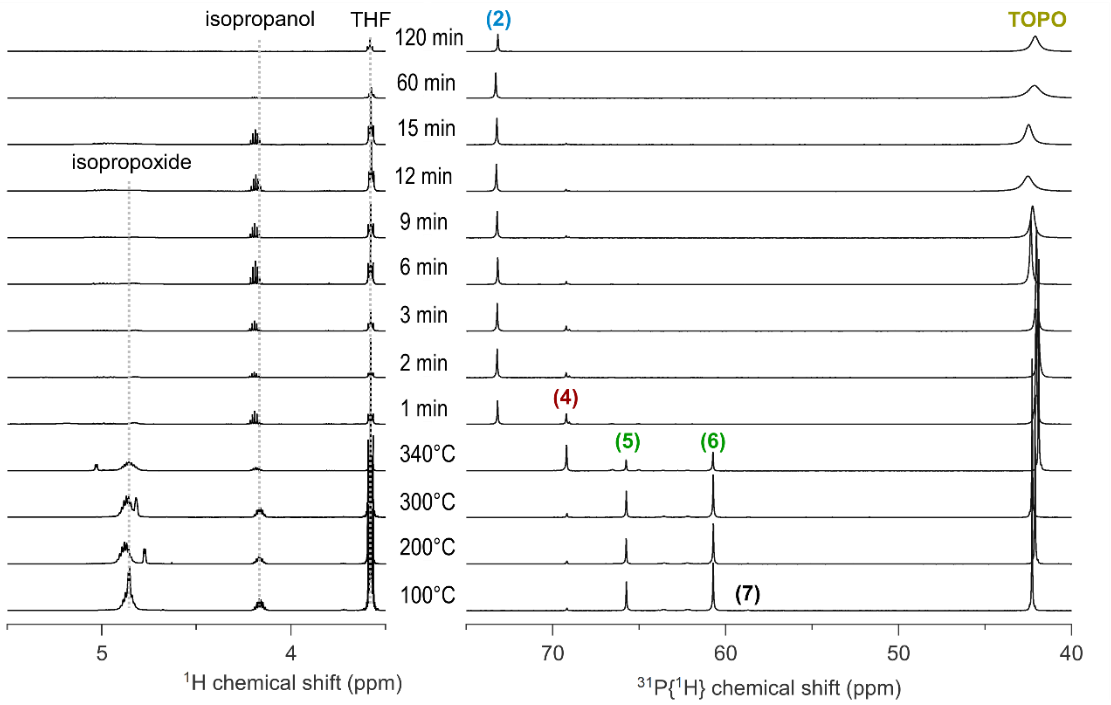

Figure S20. ^{1}H and ^{31}P NMR of the reaction mixture with 1 equivalent of $ZrCl_4$.2THF in C_6D_6. Aliquots were taken at different temperatures during the ramp and at different times at the final reaction temperature of 340 °C.

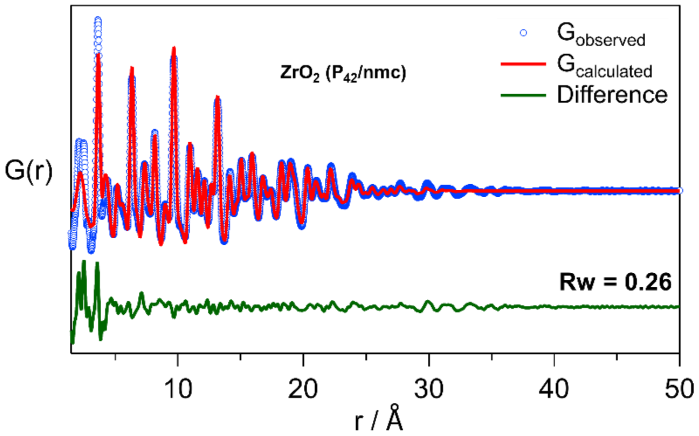

Parameter	Refined Value
Scale	0.6
a(Å)	3.62
c(Å)	5.22
Uiso O($Å^2$)	0.029
Uiso Zr($Å^2$)	0.008
psize(Å)	34.7

Figure S21. PDF fit for the reaction crude product after 90 minutes of the reaction with the tetragonal zirconia (P_{42}/nmc) single-phase model. Refined parameters are indicated.

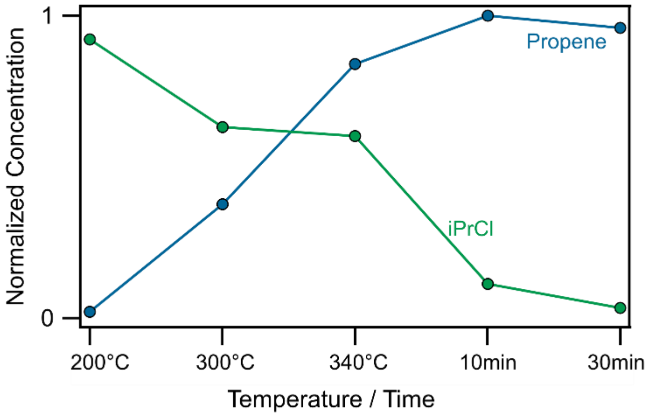

Figure S22. The concentration of propene and isopropyl chloride in the gas phase during the decomposition of *i*PrCl in presence of TOPO and $ZrCl_4$ estimated with GC-FID.

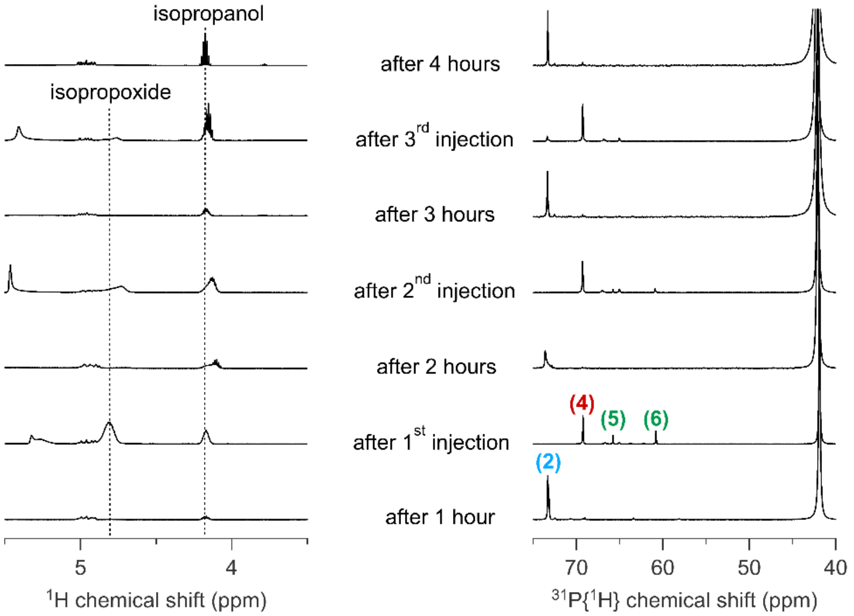

Figure S23. ^{1}H and ^{31}P NMR of the reaction mixture in C_6D_6 before and 30 seconds after each injection. The amount of $ZrCl_4$.2TOPO left after every hour was calculated from ^{31}P NMR and is respectively 0.75, 0.59, 0.50, and 0.36.

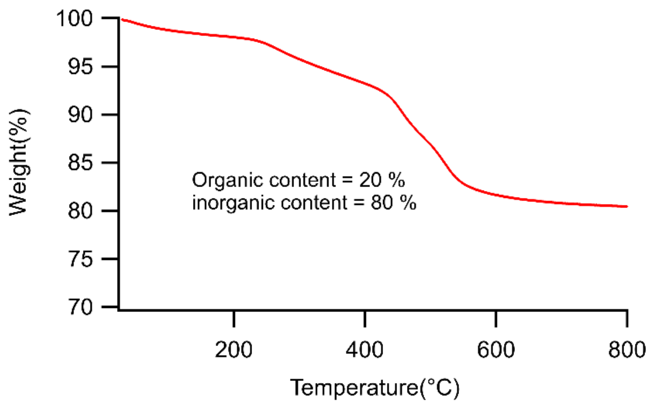

Figure S24. TGA analysis for the final product after seeded growth. Organic and inorganic content is indicated.

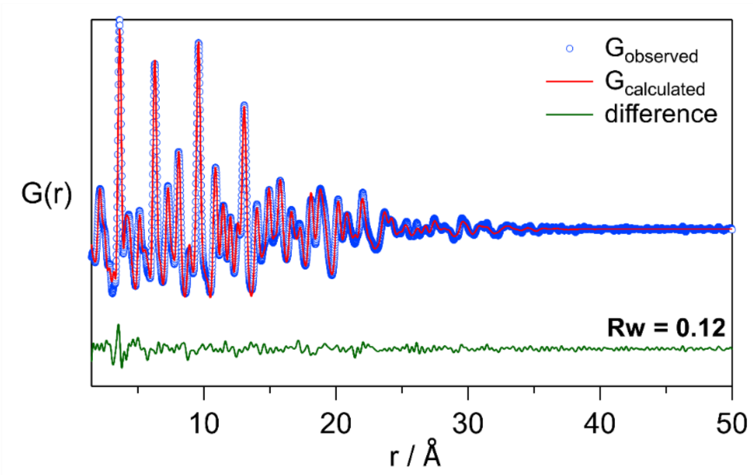

Parameter	Refined Value
Scale	1.1
a(Å)	3.60
c(Å)	5.18
Uiso O(Å^2)	0.044
Uiso Zr(Å^2)	0.008
δ_2(Å^2)	3.59
psize(Å)	41.1

Figure S25. PDF fit for the purified product with the tetragonal zirconia (P_{42}/nmc) single-phase model. Refined parameters are indicated.

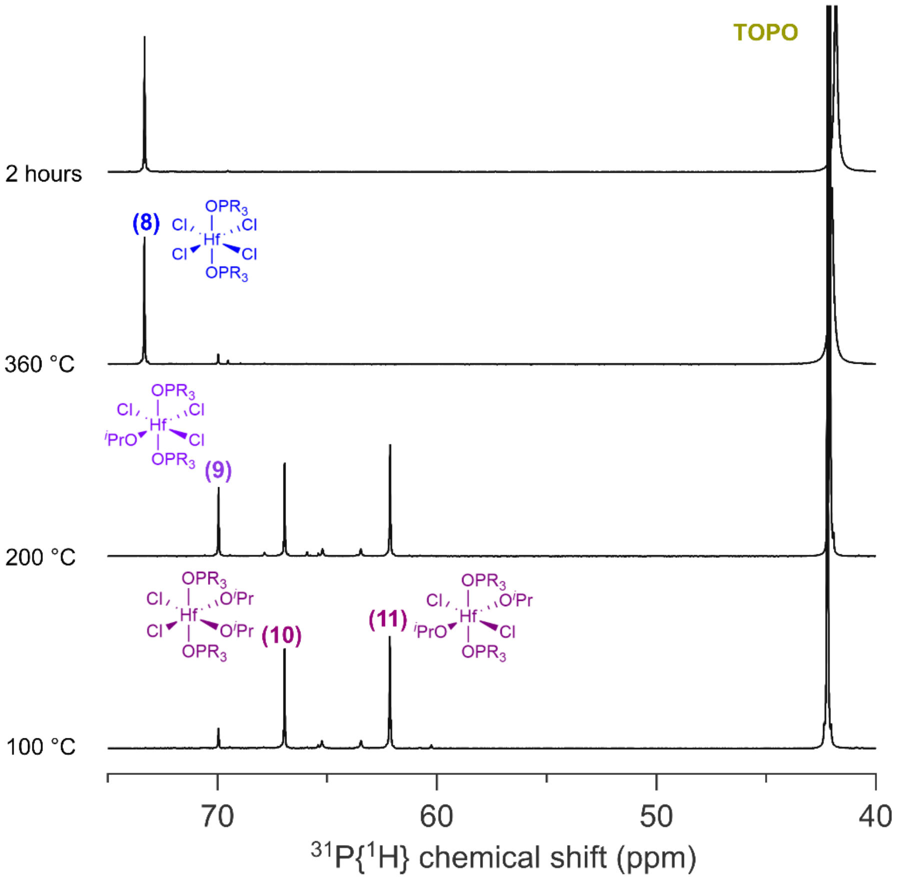

Figure S26. ^{31}P NMR of the reaction mixture with **1 : 1 equivalent of $HfCl_4$: Hf(O*i*Pr)$_4$.*i*PrOH** in C_6D_6. Aliquots were taken at different temperatures and 2 hours after reaching 360 °C.

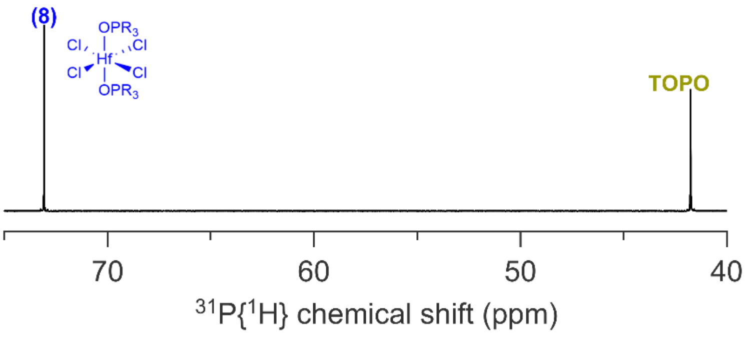

Figure S27. ^{31}P NMR of $HfCl_4$ dissolved in C_6D_6 with four equivalents of TOPO.

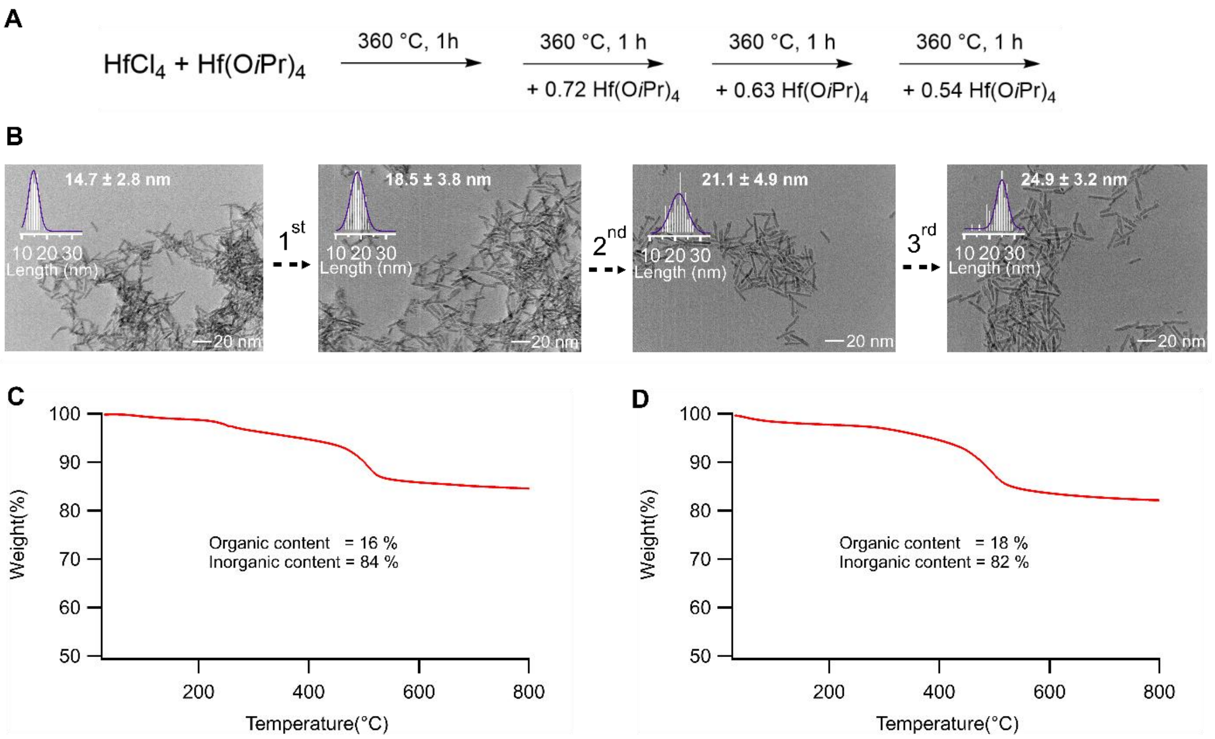

Figure S28. (A) Scheme showing the Hf(O*i*Pr)$_4$.*i*PrOH injection strategy to increase particle size and yield. **(B)** TEM and histogram of particles before and after each injection. The average particle length is indicated. The width of the nanorod is 2-3 nm. **(C)** TGA analysis for the final product after two-hour synthesis. Organic and inorganic content is indicated **(D)** TGA analysis for the final product after seeded growth. Organic and inorganic content is indicated.

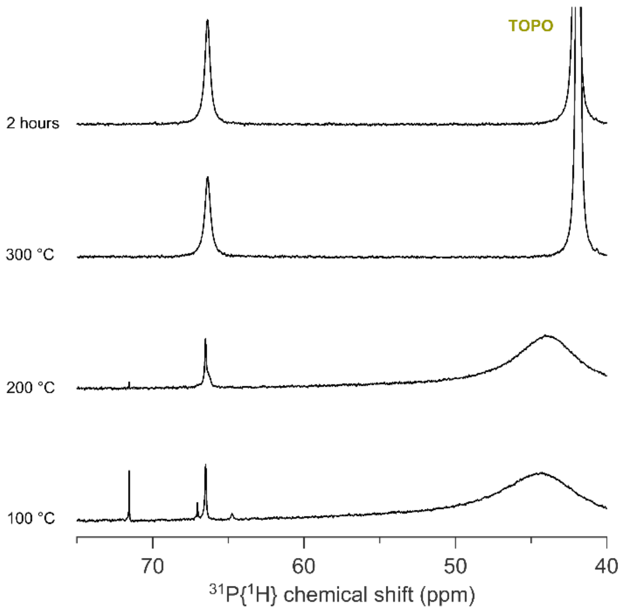

Figure S29. ^{31}P NMR of the reaction mixture with **1 : 1 equivalent of TiCl$_4$: Ti(O*i*Pr)$_4$** in C_6D_6. Aliquots were taken at different temperatures and 2 hours after reaching 300 °C.

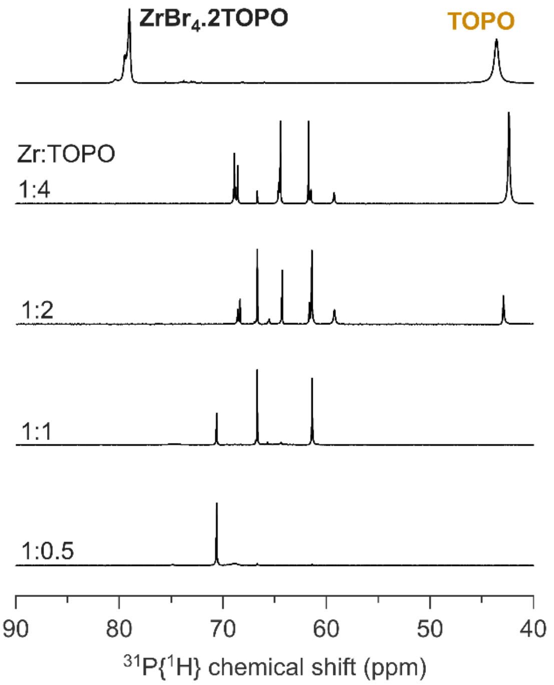

Figure S30. (A) Reaction scheme for the titration of a 1 : 1 mixture of $ZrBr_4$: Zr(O*i*Pr)$_4$.*i*PrOH with TOPO in C_6D_6 at room temperature. The reference spectrum for $ZrBr_4$.2TOPO is also shown.

Table S1 For the optimized structures, we present the unscaled, computed (using TEPO) ^{31}P shift (σ), the scaled computed shift (δ) and the experimentally measured shift (using TOPO)**.**

Structure	Computed σ	^{31}P shifts Computed δ (ppm)	Experimental δ (ppm)	Goodness of fit (difference)
TEPO	340.40	41.4	42	-0.60
$ZrCl_2$(O*i*Pr)$_2$.2TEPO (trans)	318.34	62.9	61.5	1.40
$ZrCl_2$(O*i*Pr)$_2$.2TEPO (cis)	315.10	66.1	66.4	-0.30
$ZrCl_3$(O*i*Pr).2TEPO	311.18	69.9	69.2	0.70
$ZrCl_4$.2TEPO (trans)	307.14	73.8	74.5	-0.70
$ZrCl_4$.TEPO.THF (trans)	301.44	79.4	79.8	-0.40

Table S2. For the optimized structures, we present the unscaled, computed (using TEPO) ^{1}H shift (σ), the scaled computed shift (δ) and the experimentally measured shift (using TOPO)**.**

Structure	Protons	Computed σ	^{1}H shifts Computed δ (ppm)	Experimental δ (ppm)	Goodness of fit (difference)
$ZrCl_2$(O*i*Pr)$_2$.2TEPO (cis)	Cα (O^{i}Pr)	26.86	4.60	4.20	0.40
$ZrCl_2$(O*i*Pr)$_2$.2TEPO (trans)	Cα (O^{i}Pr)	26.94	4.50	4.20	0.30
$ZrCl_4$.2THF (trans)	Cα (THF)	26,69	4.75	4.63	0.12
$ZrCl_4$.2THF (trans)	Cβ (THF)	29.33	2.24	2.16	0.08
$ZrCl_4$.(THF)(TEPO) (trans)	Cα (TPPO)	28.97	2.58	2.12	0.46
$ZrCl_4$.(THF)(TEPO) (trans)	Cα (THF)	26.73	4.70	4.63	0.07
$ZrCl_4$.2TEPO (trans)	Cα (TPPO)	29.19	2.37	2.13	0.24

Table S3. Refined values after fitting the reaction crude product after 90 minutes of the reaction with a dual-phase model of the tetragonal zirconia (P_{42}/nmc) and the $ZrCl_4$.2TPPO complex **(2).**

Parameter	Refined Value<br>Phase 1 - ZrO_2(P_{42}/nmc)	Refined Value<br>Phase 2- $ZrCl_4$.2TPPO(2)
Scale	0.72	0.54
a(Å)	3.63	-
c(Å)	5.22	-
Uiso O(Å^2)	0.06	0.02
Uiso Zr(Å^2)	0.01	0.011
Uiso Cl(Å^2)	-	0.001
Uiso C(Å^2)	-	0.02
Uiso P(Å^2)	-	0.001
δ_2(Å^2)	3.13	-
psize(Å)	40	-

Table S4. Refined values after fitting the purified product (after three injections) with the tetragonal zirconia (P_{42}/nmc) single-phase model.

Parameter	Refined Value
Scale	1
a(Å)	3.60
c(Å)	5.19
Uiso_O(Å^2)	0.046
Uiso_Zr(Å^2)	0.007
δ_2(Å^2)	3.8
psize(Å)	53.3
